# Supplementary figures and images for: Pax6 organizes the anterior eye segment by guiding two distinct neural crest waves
Source: PLoS Genet. 2020 Jun 17;16(6):e1008774. doi: 10.1371/journal.pgen.1008774 (PMC7323998; doi:10.1371/journal.pgen.1008774)

S1 Figure

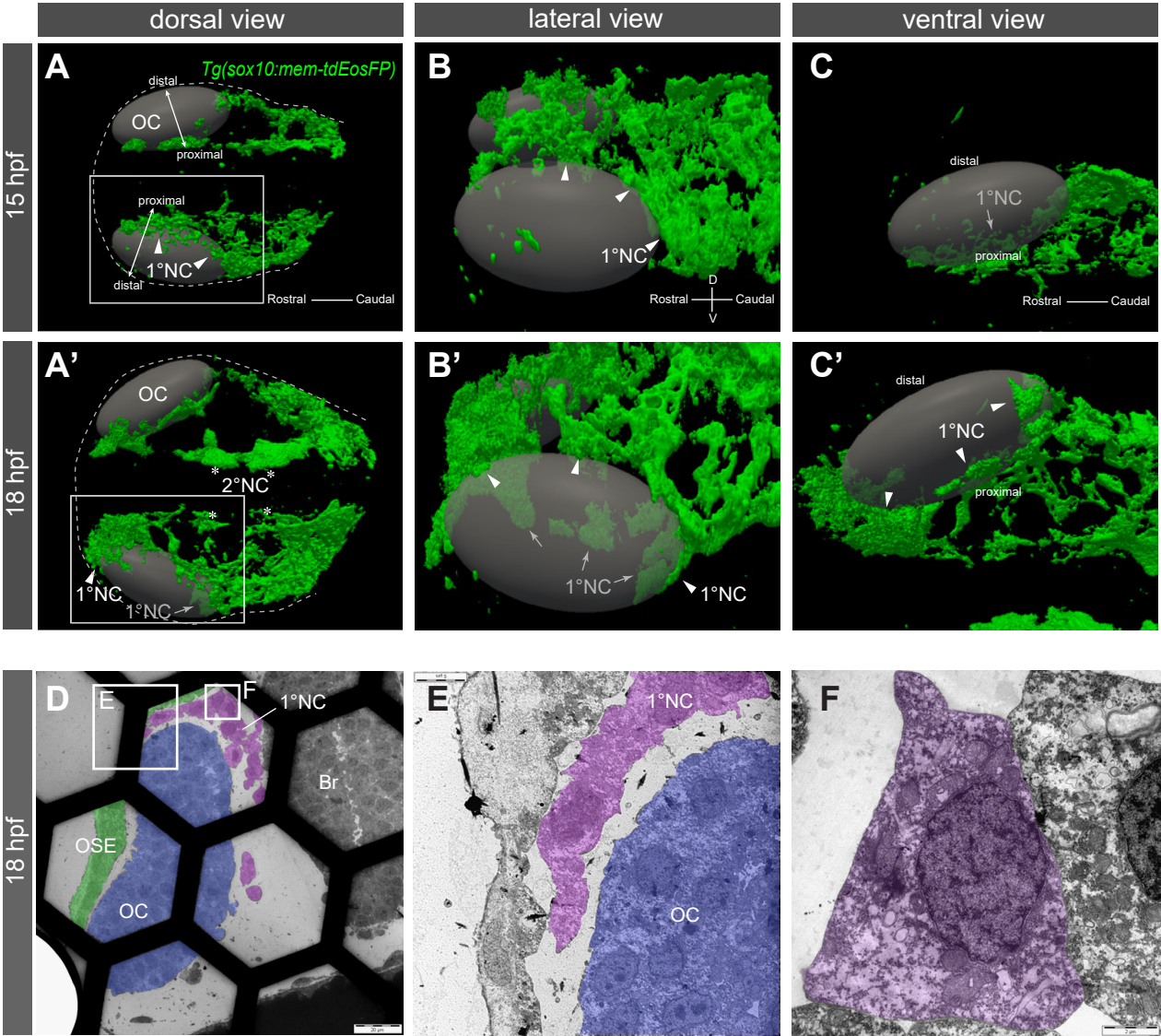

Supplement: S1 Fig — (A-C’) 3D rendered images of a Tg(sox10:mem-tdEosFP) embryo are shown from three different angles: dorsal views (A-A’), lateral views (B-B’) and ventral views (C-C’). Anterior, left. Two selected time points (A-C: 15 hpf; A’-C’: 18 hpf) are shown. The grey spheroid represents the optic cup (OC). One side of the eye (rectangular region) in the dorsal view (A-A’) is highlighted in lateral (B-B’) and ventral views (C-C’). (A-C) At 15 hpf, 1°NC cells start covering the optic cup from its posterior end and its proximal side (arrowheads, A). Only a small dorsal portion of the eye is covered by 1°NC cells (arrowheads in B, arrow in C), while the ventral half of the eye is not covered with 1°NC cells (C). The arrow in C points at 1°NC cells on the dorsal side of the eye. (A’-C’) By 18 hpf, 1°NC cells have reached the rostral end of the optic cup (arrowhead in A’), with the proximal side of the eye covered roughly halfway (arrows in B’ and arrowheads in C’). Importantly, 1°NC cells exclusively migrate over the proximal side of the eye (arrows, B’) and do not migrate over the lens side (distal side; arrowheads, B’). Note that 2°NC cells in clusters (asterisks in A’) have not yet reached the eye at 18 hpf. (D-F) Ultrastructure of 1°NC cells (magenta). (D) 1°NC cells are located on the proximal side of the optic cup (blue; transverse view at 18 hpf). The regions indicated by squares are shown in E and F. Black honey comb pattern is from the grid. (E) 1°NC cells form a mono-layered sheet. (F) A 1°NC cell (coloured in magenta) show a large cell-cell contact plane with neighbouring 1°NC cells. OSE: optic surface epithelium; Br: brain. (PDF) [file pgen.1008774.s011.pdf]

S2 Figure

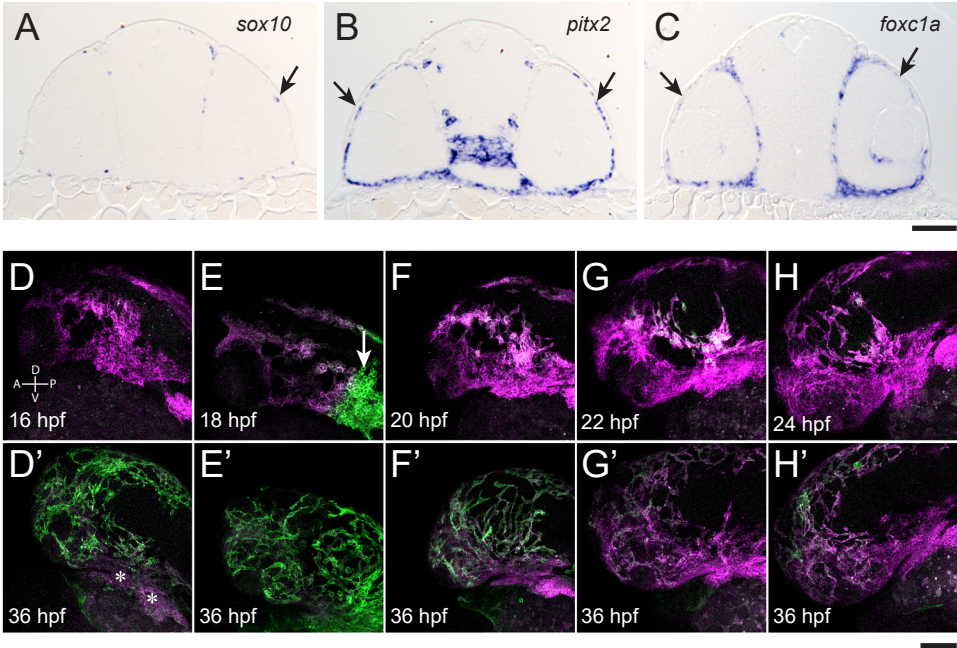

Supplement: S2 Fig — (A-C) in situ gene expression analysis of sox10 (A), pitx2 (B) and foxc1a (C) in WT embryos at 28 hpf. Arrows indicate peri-ocular mesenchymal cells. Scale bar: 50 μm. (D-H’) Photoconversion of mem-tdEosFP and recovery of green fluorescence for visualization of de novo sox10 reporter expression. At the indicated stages, mem-tdEosFP was switched from green to red (magenta) by illumination with 400-nm light (D-H). Continued mem-tdEosFP expression was scored by reappearance of green fluorescence at 36 hpf (D’-H’). Recovery of green mem-tdEosFP fluorescence in the eye at 36 hpf was noticed in embryos photoconverted until 20 hpf (D-F’). Photoconversion at 22 or 24 hpf showed recovery of green fluorescence in the head but not in the eye region (G’-H’), indicating that 2°NC cell derivatives which have migrated into the eye have turned off sox10 transgene expression by 22 hpf. In contrast, sox10 reporter expression in NC derivatives has ceased in the pharyngeal arches at 16 hpf (asterisks, A’) as well as in 1°NC on the proximal side of the retina. Green NC cells in panel E (white arrow) are un-photoconverted cells. Scale bar: 40 μm. (PDF) [file pgen.1008774.s012.pdf]

S3 Figure

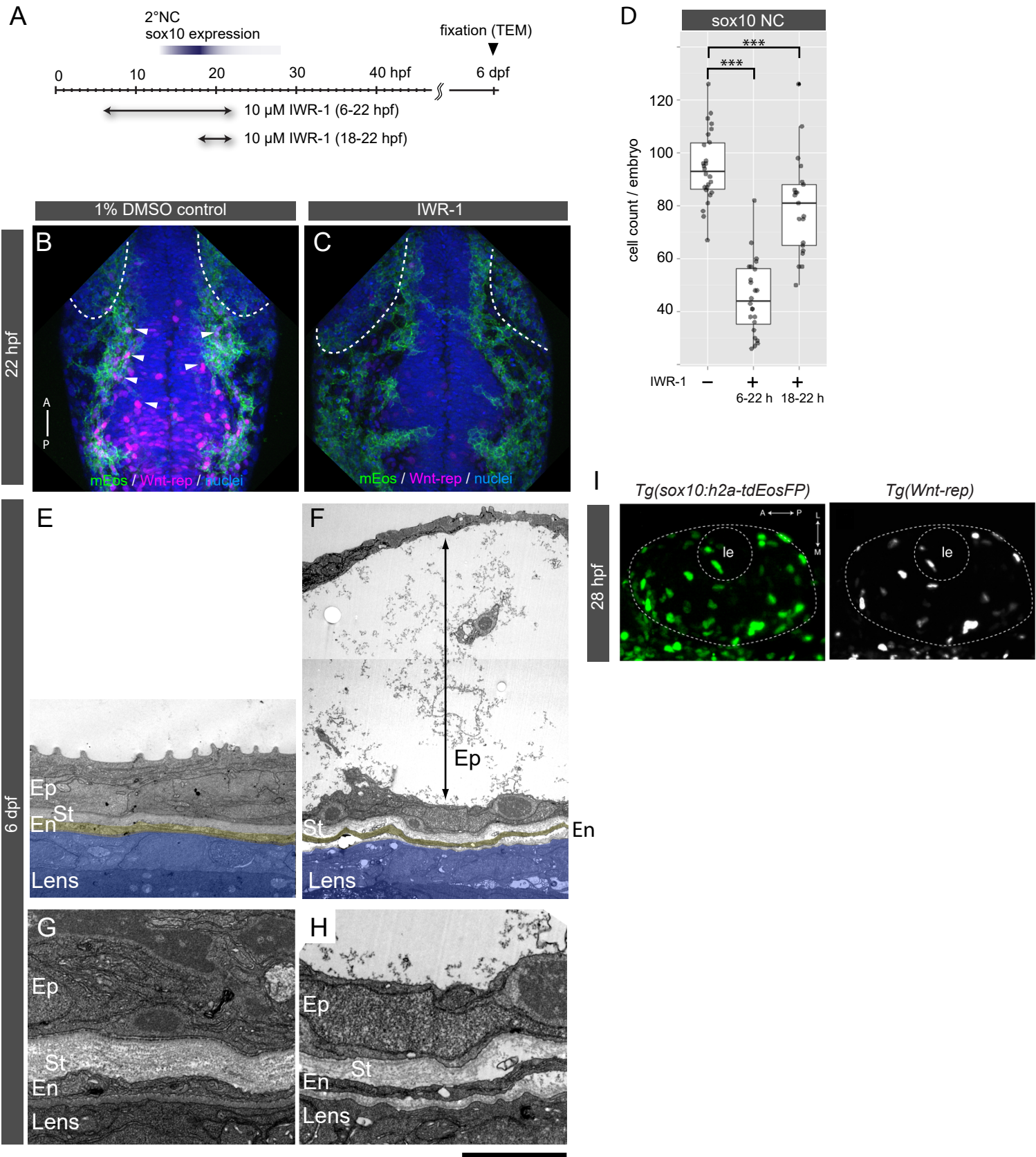

Supplement: S3 Fig — (A): Embryos were treated with either 1% DMSO alone or the Wnt inhibitor (10 μM IWR-1, 1% DMSO) from 6 to 22 hpf or 18 to 22 hpf. The phase of active sox10 expression in 2°NC cells is indicated. (B-D): sox10:mem-tdEosFP (green) and Wnt-rep (magenta) expressing embryos were exposed to solvent (B) or Wnt inhibitor (C) from 6 to 22 hpf and fixed and counter-stained with DAPI (blue). Inhibition of canonical Wnt signalling abolished the expression of the Wnt-rep (magenta, arrowheads, B) and reduced the expression of mem-tdEosFP (C). Quantification of endogenous sox10 mRNA positive cells (D) reveals a significant decrease of sox10 expressing cells anterior to the otic vesicle at 22 hpf for both exposure windows (p< 0.001, n = 24 embryos, 6–22 hpf, n = 21 embryos, 18–22 hpf) compared to controls (n = 26). (E-H): Ultrastructure of the corneal endothelium at 5 dpf in control (E, G; n = 3 embryos) and IWR-1 treated embryos (6–22 hpf, F, H; n = 3 embryos). Transverse sections through the centre of the lens. Inhibition of Wnt signalling caused formation of an abnormal corneal epithelium (Ep) with the inner and outer epithelial layers being separated by a large oedema. No effect on the formation of the corneal endothelium (En) or the thickness of the stroma (St) was notedic (G, H; n = 3). Orientation of embryos: B-C: anterior up, view onto dorsal. Scale bar: B-C: 70 μm; E-F: 6 μm; G-H: 2 μm (I) Dorsal view of the eye from Tg(sox10:h2a-tdEosFP;Wnt-rep) line at 28 hpf. Anterior left. Approximately 50% of NC cells (23/47 cells) in the distal side of the eye (green, left panel) express Wnt reporter (right panel). le: lens. (PDF) [file pgen.1008774.s013.pdf]

S4 Figure

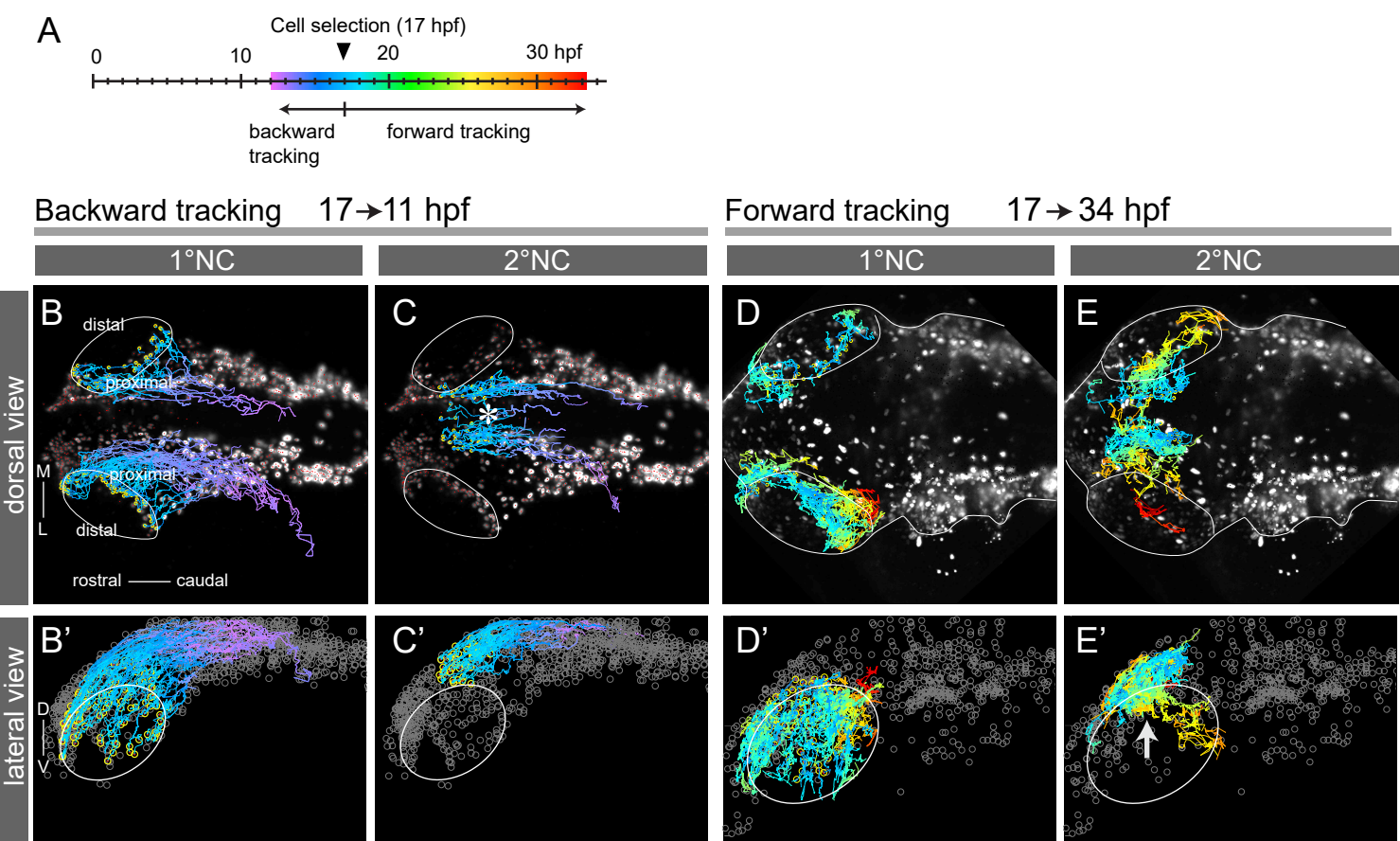

Supplement: S4 Fig — Tracking analysis of 1°NC and 2°NC cells from a second embryo double transgenic for Tg(sox10:h2a-tdEosFP) and Tg(Wnt-rep) that was imaged essentially under the same conditions as the one shown in Fig 2. Two groups of h2a-tdEosFP-positive NC cells were selected at 17 hpf for systematic tracking as shown in Fig 2; 1°NC cells that were already in contact with the optic cup (112 cells; yellow circles in B, B’, D, D’) and 2°NC cells that formed the temporal cell cluster next to the diencephalon and mesencephalon (62 cells; yellow circles in C, C’, E, E’). B-E, dorsal projection with rostral left; B’-E’, lateral projection with dorsal up. Tracks are shown with a temporal colour code shown in the panel A. 2°NC cells at the dorsal edge of the eye originated from the diencephalon and mesencephalon (asterisk, C) and migrated into the anterior chamber of the eye from the dorsal side of the retina (arrow in E’). Scale bar: 100 μm. (PDF) [file pgen.1008774.s014.pdf]

S5 Figure

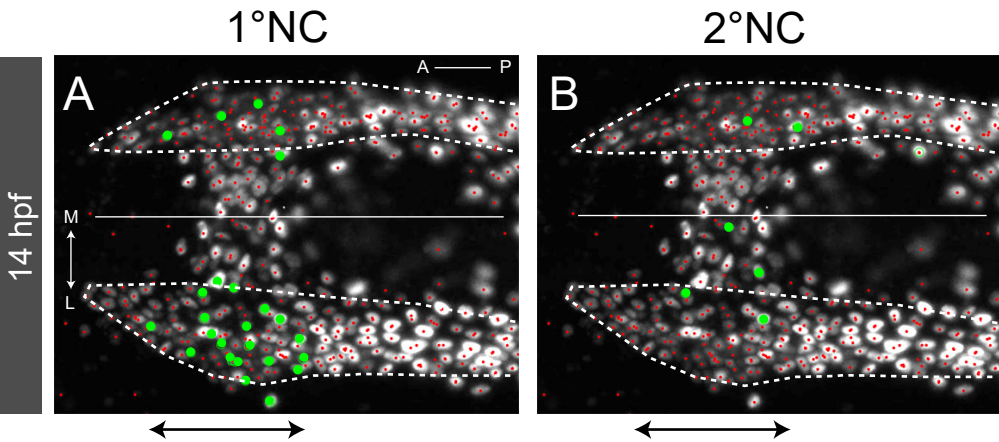

Supplement: S5 Fig — (A-B) Backward tracking analysis of 1°NC and 2°NC cells in a Tg(sox10:h2a-tdEosFP) embryo at 14 hpf. At 17 hpf, H2a-tdEosFP-positive cells that were already in contact with the optic cup were selected for 1°NC tracking (n = 112 cells) and those found at the diencephalic and mesencephalic regions for 2°NC tracking (n = 62 cells). Positions of 1°NC (A) and 2°NC cells (B) at 14 hpf are shown in green, merged over the dorsal maximum projection view. Red dots are the centres of the nuclei. Anterior is left. (A) 1°NC cell nuclei (green spots, 25 out of the selected 112 cells) are distributed in the anterior part of the embryo, in relatively narrow lateral regions along the anterior-posterior axis (less than 10-cell diameters, horizontal double-headed arrow). Along the mediolateral axis, 1°NC cells are found laterally within the lateral NC stripes (stippled line enclosed area). Within each lateral NC stripe, 1°NC cells show no obvious mediolateral bias. (B) In comparison, 2°NC cell nuclei (6 green spots in B) are found biased toward the medial part of the embryo within the narrow anteroposterior region in which 1°NC cells are found (horizontal double-headed arrow). (PDF) [file pgen.1008774.s015.pdf]

S6 Figure

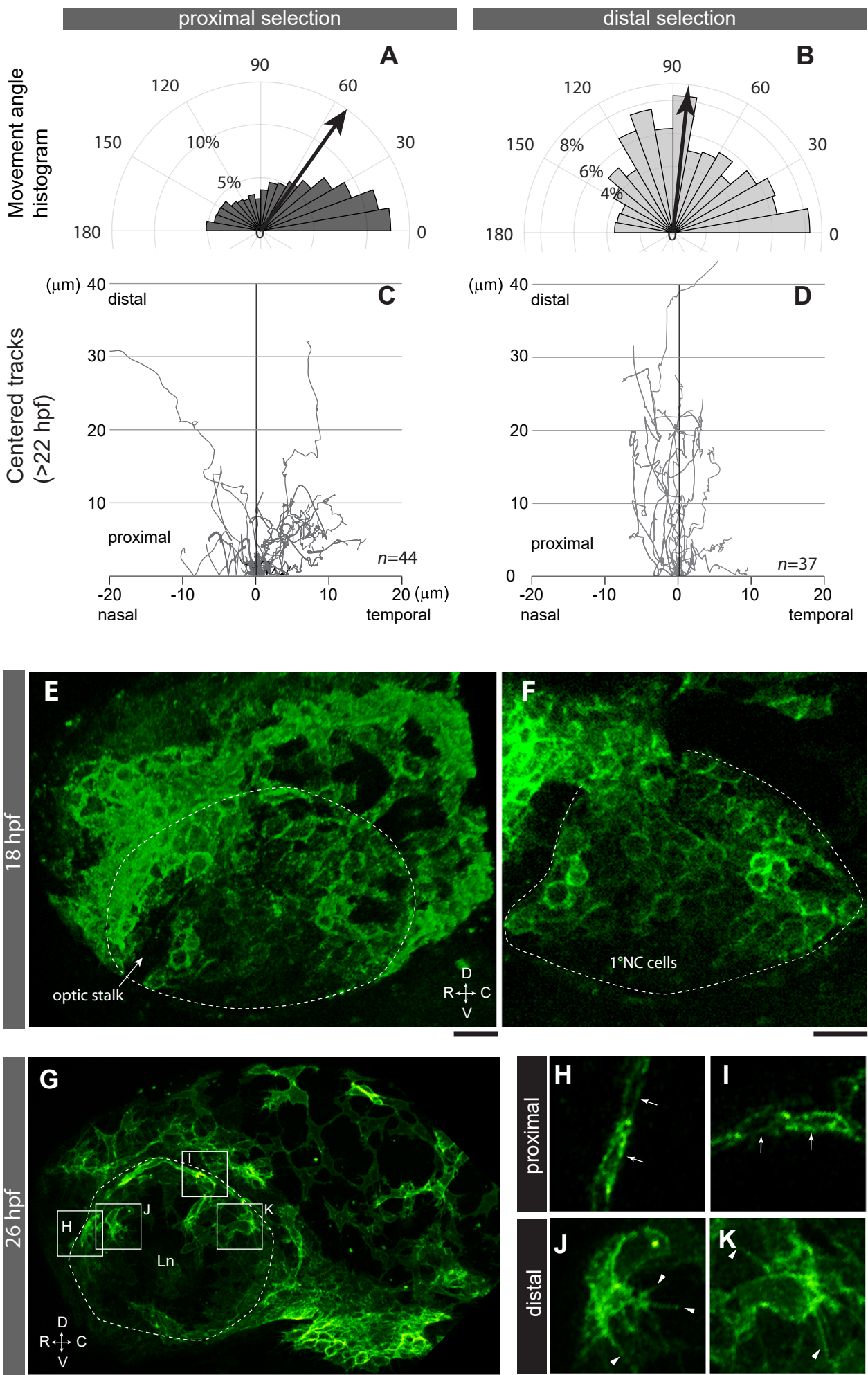

Supplement: S6 Fig — (A, B) Movement angle histogram for NC cells destined for proximal (A, 1°NC cells; n = 44) and distal (B, 2°NC cells; n = 37) sides of the eye. Movement trajectories of an individual NC cell were projected on a horizontal plane and the angle relative to the anteroposterior axis was measured every 10 time points (every 3 min 24 s). Each bin is 10°. Black arrows show median direction (56.5° and 83.6° for proximal and distal selection, respectively). Wallraff test of angular distances between two groups showed significant difference, p-value < 2.2x10^-16^. (C, D) Centred tracks starting from 22 hpf for proximal (C, 1°NC; n = 44) and distal (D, 2°NC; n = 37) destined NC cells. The starting position is set to 0 (bottom centre). (C) NC cells on the proximal side of the eye show moderate distal-oriented movement after 22 hpf. (D) In contrast NC cells destined for the distal side of the eye show higher displacement toward the distal end. (E-K) Cell morphology of 1°NC and 2°NC cells. (E-F) A 18-hpf embryo from Tg(sox10:mem-tdEosFP), highlighting NC cells in green. (E) 3D-overview of the optic cup region (stippled circle). (F) Projected focal planes for the proximal side of the eye (stippled region). Note the cuboidal or hexagonal cell shape of 1°NC cells retaining large areas of cell-cell contact with neighbouring cells. (G-K) Overview of a 26-hpf Tg(sox10:mem-tdEosFP) embryo (G, maximum projection) and magnified single cell morphology of NC cells in a single focal plane for proximal (H-I) and distal (J-K) ocular NC cells. Squares in G show individual locations of magnified cells in H-K. 1°NC cells appear flat and rectangular (arrows), while 2°NC cells show mesenchymal morphology with filopodia-like cellular protrusions (arrowheads). Orientation: dorsal (D), ventral (V), rostral (R) and caudal (C); Ln: lens; Scale bars: 20 μm. (PDF) [file pgen.1008774.s016.pdf]

S7 Figure

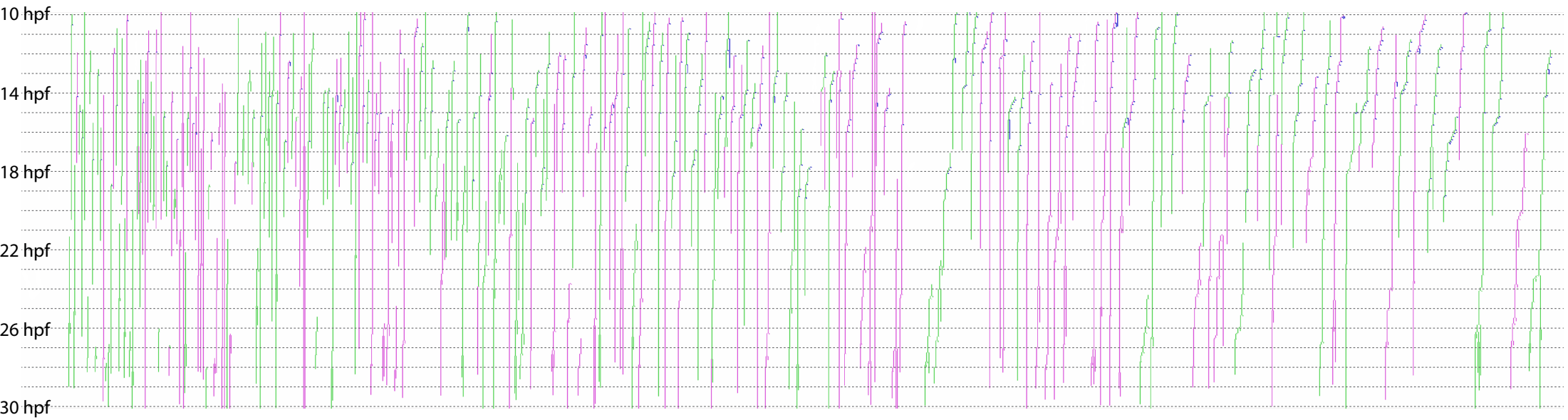

Supplement: S7 Fig — Lineages for each of the proximal (magenta) or distal (green) destined group are shown in tree diagram. Each branch corresponds to a cell division. Incomplete short tracks are shown in blue. (PDF) [file pgen.1008774.s017.pdf]

S8 Figure

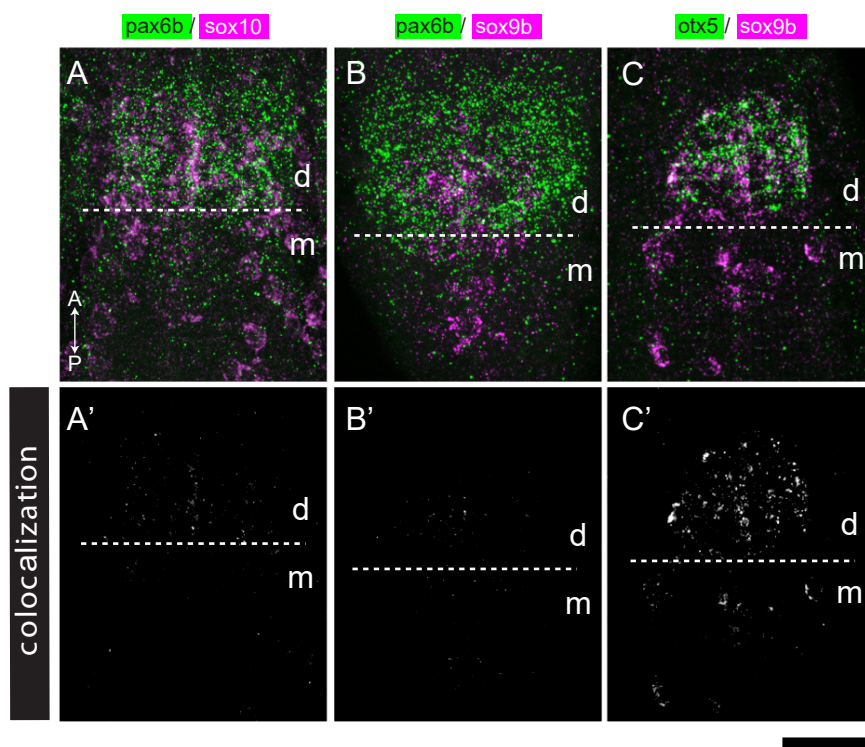

Supplement: S8 Fig — Wildtype embryos at 18 hpf were examined for the indicated combinations of diencephalic/mesencephalic marker genes by fluorescence double in situ hybridisation. Anterior top, dorsal views. The boundary between diencephalon (d) and mesencephalon (m) is indicated by a stippled horizontal line. (A-B) In dorsal views, both sox10 and sox9b appear co-expressed with pax6b that is expressed in the posterior diencephalon. However, co-localization analysis showed neither sox10- nor sox9b-positive cells co-express pax6b (A’ and B’). The posterior end of pax6b expression marks the boundary between diencephalon and mesencephalon (stippled line). (C, C’) sox9b-positive cells (magenta) in the diencephalon co-express the epiphysis marker gene, otx5 (green). Scale Bar: (A-C, A’-C’) 50 μm. (PDF) [file pgen.1008774.s018.pdf]

S9 Figure

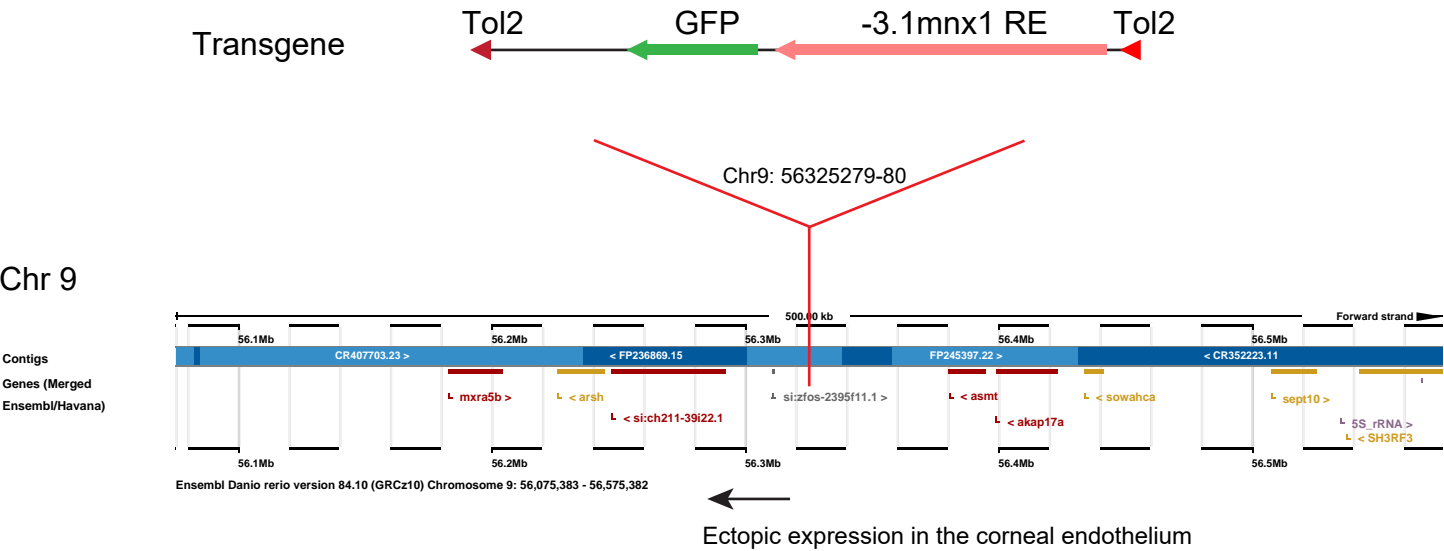

Supplement: S9 Fig — A genomic DNA library was prepared from 50 embryos and paired-end reads (2×50 nucleotides) were obtained with an Illumina Hiseq1500 sequencer. Two insertion sites, one in chromosome 7 and another in chromosome 9, were identified. By outcrossing into wild type, the insertion site mapped to the end of chromosome 9 on the minus strand (Chr9: 56325279–80, GRCz10) was found responsible for the ectopic corneal endothelium expression of the GFP reporter. (PDF) [file pgen.1008774.s019.pdf]

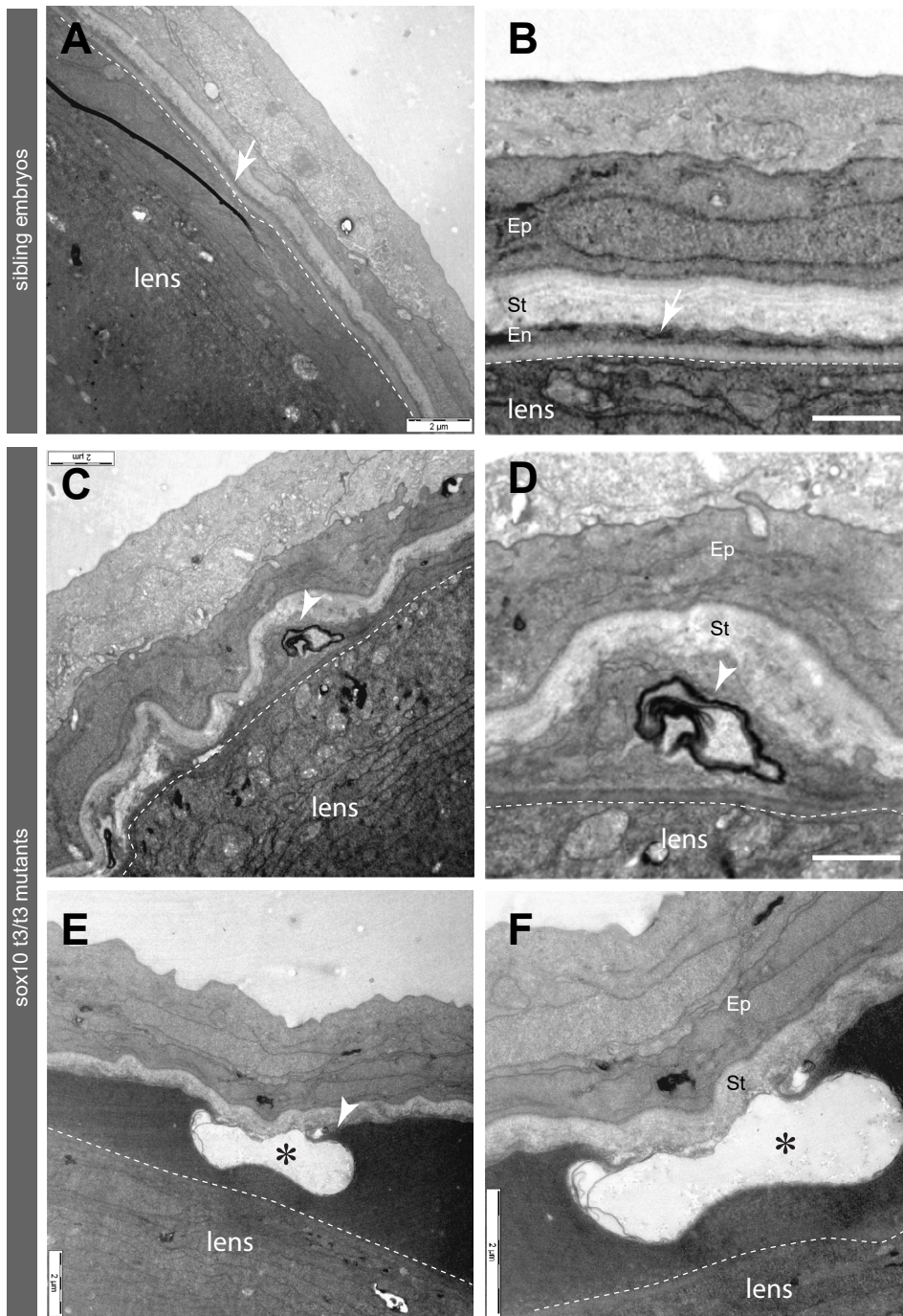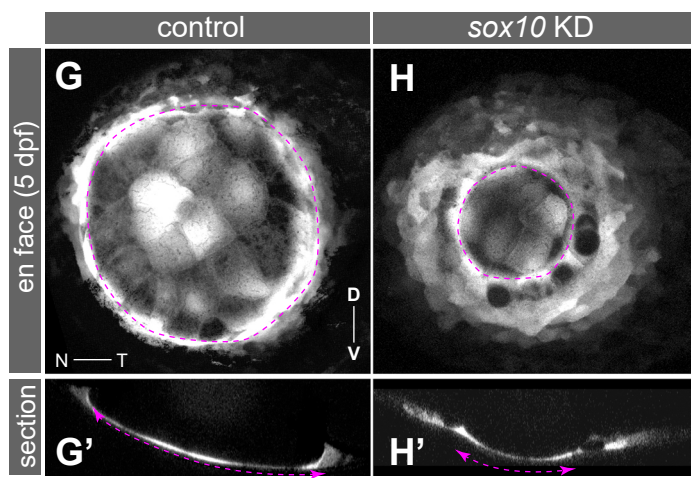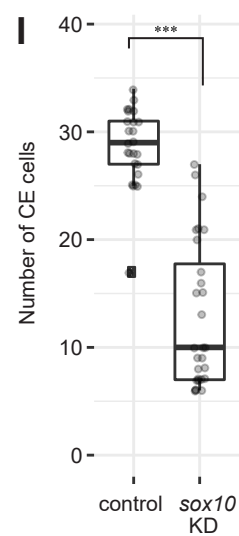

G: n=25 embryos  
H: n=28 embryos

Supplement: S10 Fig — (A-F) Ultrastructure analysis of the cornea (arrow) from sox10t3/+ or sox10+/+ sibling embryos (A-B, n = 2 embryos) and sox10t3/t3 homozygous mutants (C-F, n = 4). Low magnification overviews (A, C and E) and respective magnified views (B, D and F) are shown. Stippled line demarcates the lens. sox10t3/t3 homozygous mutants showed vacuolated thick layer of cells (C-D) beneath the corneal stroma (St). Thinning of the same layer with oedema (asterisk) was also observed (E-F). Scale bar: 1 μm (B, D); (G-I) Morpholino knockdown of sox10 caused abnormal AS with reduced number of the corneal endothelium. (G-H’) At 5 dpf the corneal endothelium (grey) forms a monolayer of cells over the lens, which is surrounded by a ring of annular ligament cells (stippled magenta circle) in en face views (G-H). (G’-H’) Corresponding transverse section views along the nasotemporal axis of a control and sox10-KD embryo shown in G-H. (G’-H’) Stippled double-head arc arrows (magenta) show the extent of the corneal endothelium. Note in agreement with the electron micrographs the endothelial cells appear thicker and more irregular than the control endothelial cells in G’. Orientation: nasal (N), temporal (T), dorsal (D) and ventral (V); Scale bar: 50 μm (I) Control embryos at 5 dpf have 28.6 ± 3.6 corneal endothelial cells (n = 25), whereas sox10-KD embryos show 12.8 ± 6.7 cells (n = 28). ***Welch Two sample t-test p-value = 1.3 x10^-13^ (t = 10.7, df = 42.1). (PDF) [file pgen.1008774.s020.pdf]

S11 Figure

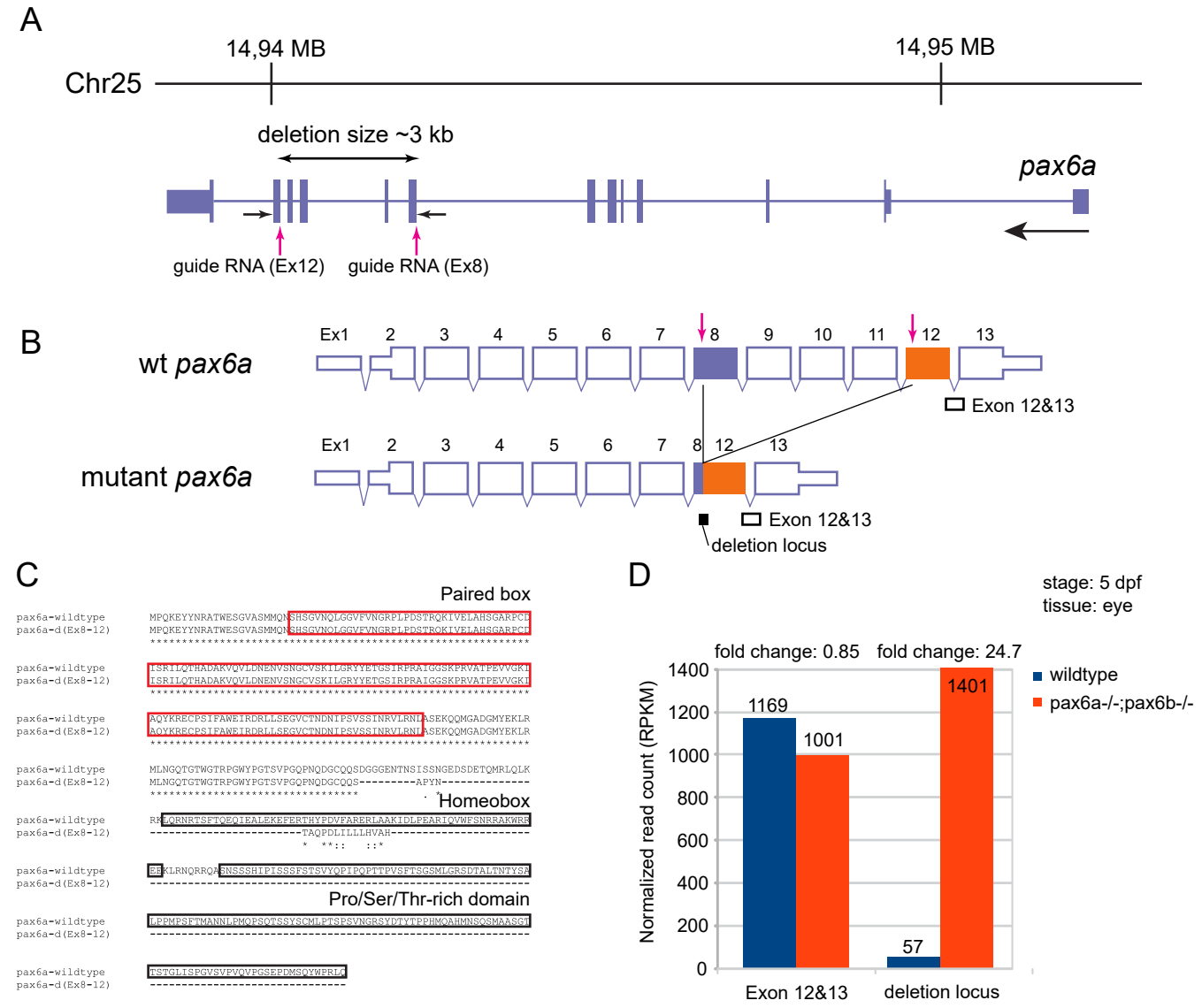

stage: 28 hpf

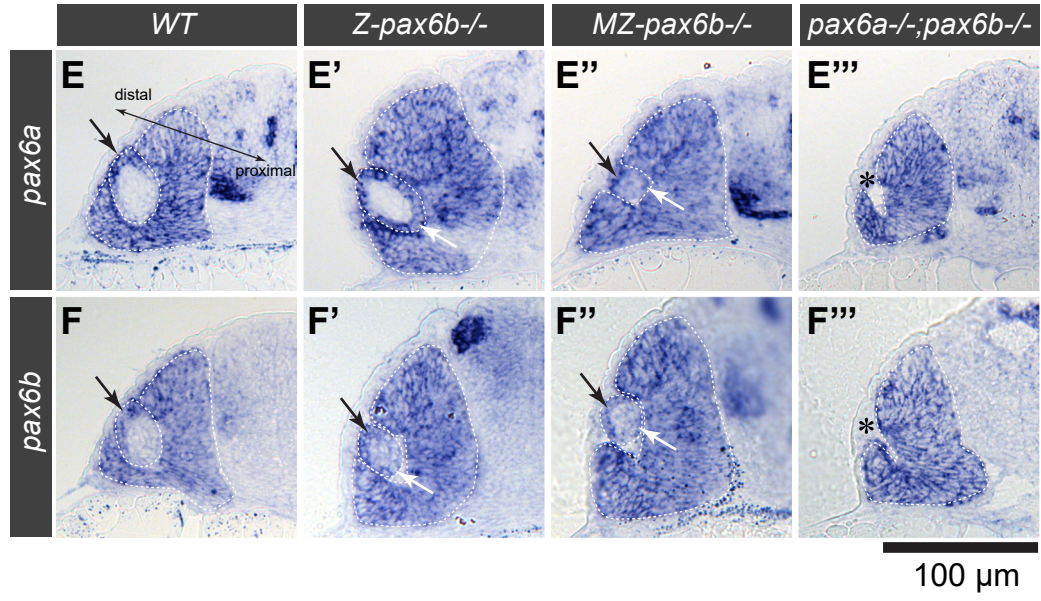

Supplement: S11 Fig — (A) The chromosome 25 pax6a locus highlighted with two guide RNA sites in exon 8 and 12 (magenta arrows). Two primer sites for identifying the genomic deletion (double headed arrow) are indicated by the small black horizontal arrows. Large black arrow: direction of transcription. (B) Schematic diagram of a predicted mutant pax6a transcript is given in parallel with the wild type transcript. Length of each exon is equalized. Magenta vertical arrows indicate the site of the two guide RNAs. (C) Predicted protein sequence of mutated pax6a locus is aligned with wild type pax6a sequence. The 3 kb genomic deletion causes a frame shift between paired box and homeobox DNA binding domains. (D) RNAseq analysis of mutant pax6a mRNAs. Transcripts in the eye of wild type and pax6a-/-;pax6b-/- double mutants at 5 dpf were isolated and processed for library construction for 50-bp paired-end RNA sequencing. Reads mapped across the deletion locus created by exon 8–12 fusion (black boxed region in B) were 1401 RPKM (reads per kilobase of transcript, per million mapped reads) for pax6a-/-;pax6b-/- double mutants, whereas only 57 RPKM were mapped here in wild type eyes (24.7-fold increase in pax6 mutants). In contrast, the number of reads mapped across the exon 12–13 boundary (white boxed region in B), which were expected to be equal between embryos from both genotypes, was comparable between the two groups (1.18-fold decrease in pax6 mutants). (E-F) in situ analysis of gene expression of pax6a (E-E”‘) and pax6b (F-F”‘) in the eye at 28 hpf with wild type (WT) embryos (E-F), zygotic (Z) pax6b-/- mutants (E’-F’), maternal-zygotic (MZ) pax6b-/- mutants (E”-F”) and pax6a-/-;pax6b-/- double mutants (E”‘-F”‘). Transverse sections at the level of the lens (or the equivalent cavity denoted by an asterisk for the lensless pax6a-/-;pax6b-/- mutants) are shown. Dorsal side is up with the distal side oriented to the left. The boundary of the eye and the lens are indicated by the stippled lines. In [file pgen.1008774.s021.pdf]

S12 Figure

A

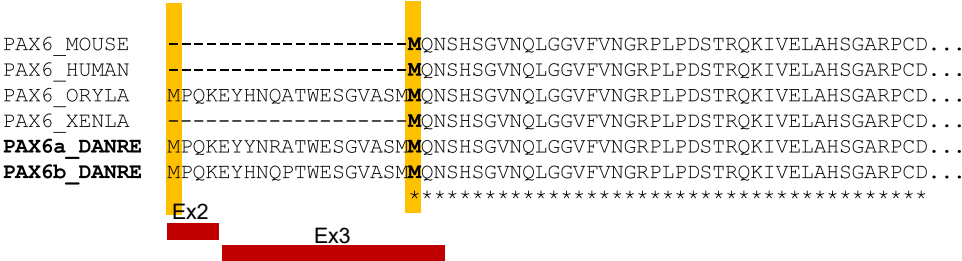

B

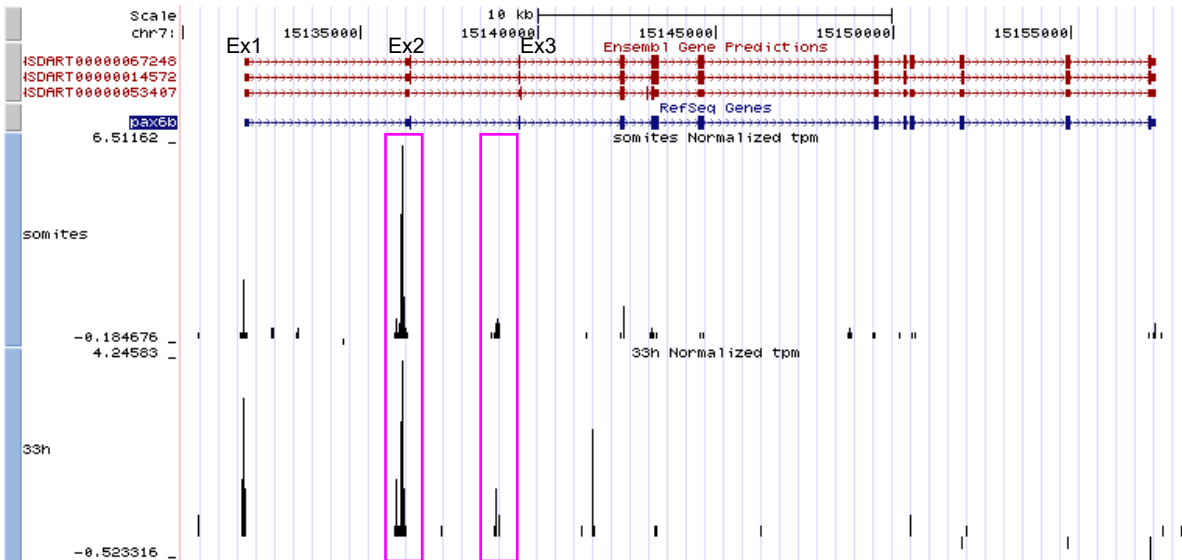

C

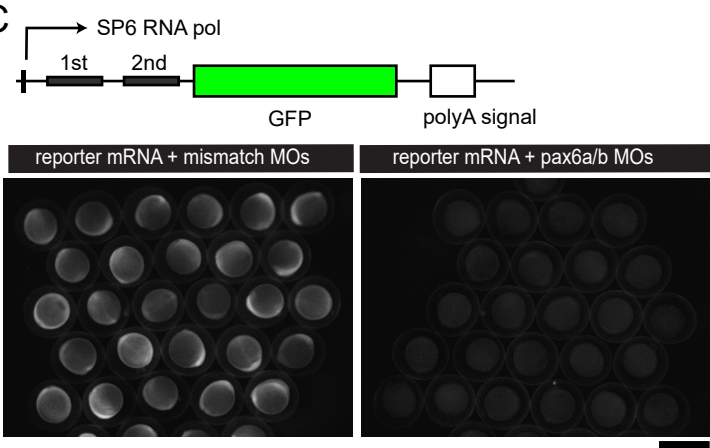

D

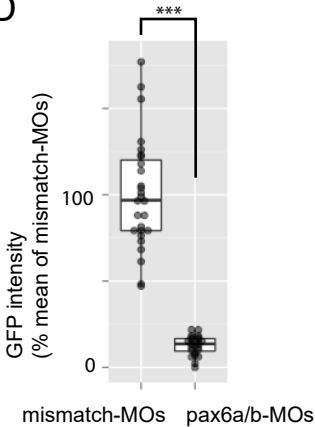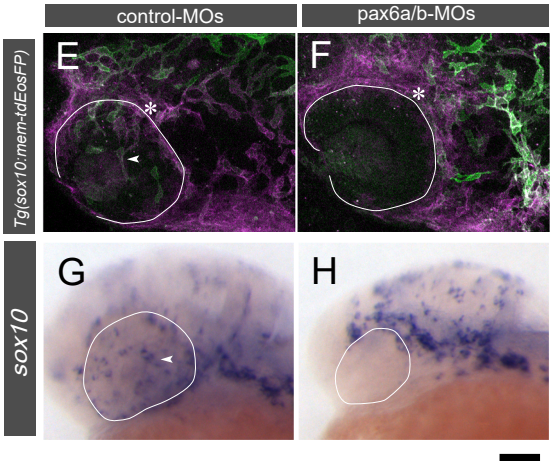

Supplement: S12 Fig — (A) Protein sequence alignment of human PAX6 and zebrafish Pax6 proteins. A part of the N-terminal region (first 87 amino acids from human PAX6) is shown. Note that zebrafish Pax6 proteins have two in-frame methionine residues (marked with orange) and the second translation start site is conserved in the human genes. Location of exon2 (Ex2) and exon3 (Ex3) for zebrafish Pax6 proteins are indicated below the sequence. (B) CAGE tag distribution for zebrafish pax6b locus [77]. Results from two stages (somitogenesis stages and 33 hpf) relevant to the anterior chamber formation are shown. Note that the two regions (magenta rectangle) corresponding to the first and second methionine of the Pax6b protein are used as transcription start sites, indicating the necessity to block both transcripts for effective knockdown of Pax6b protein. Two antisense morpholino oligonucleotides (MOs) used in this study (1ATG_pax6ab: 5’-GTTATGGTATTCTTTTTGAGGCATT-3’; 2ATG_pax6ab: 5’- ACTGTGACTGTTTTGCATCATGGAC-3’, see also Supplemental Methods) target both Pax6b transcripts as well as Pax6a. (C-D) Knockdown efficiency of pax6a/b MOs was tested on a reporter construct that harbours two MO target sites (1st and 2nd ATG region) upstream of the GFP coding sequence. mRNA of the reporter construct was synthesized by SP6 RNA polymerase and injected into one-cell stage wildtype zebrafish embryos with or without pax6a/b morpholinos (MOs). Injected embryos were raised until 10 hpf and GFP fluorescence was examined under a stereomicroscope (C) and quantified with the image analysis software ImageJ (D). pax6a/b MOs reduced GFP intensity significantly in comparison to injection of the reporter construct alone (p<0.001, D). Scale bar: 1 mm. (E-F) The Sox10 reporter mEos was converted from green to red (magenta) at 20 hpf in embryos injected with either mismatch control (E) or pax6a/b (F) MOs. This protocol led to selective labelling of 1° and 2°NC cells in magenta and green, respectively. Embryos were examine [file pgen.1008774.s022.pdf]

S13 Figure

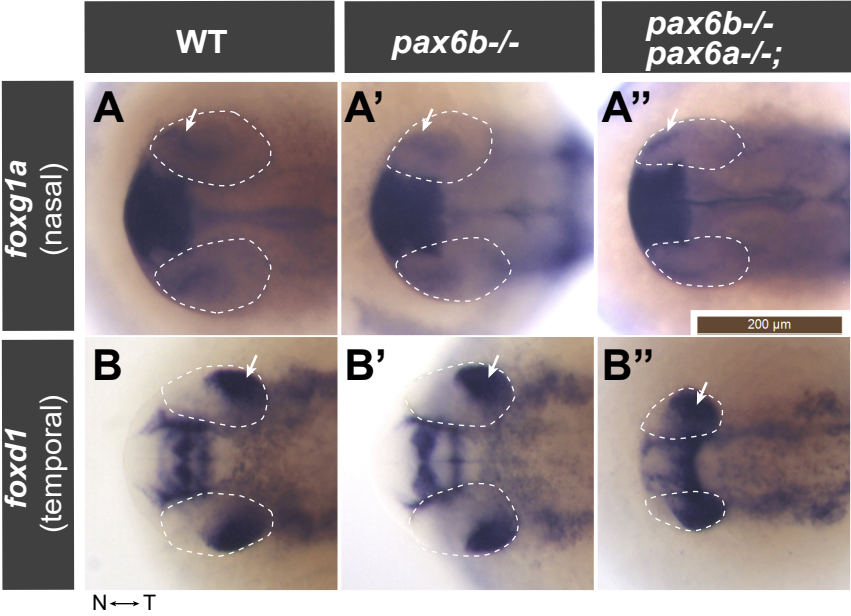

Supplement: S13 Fig — Embryos (28 hpf) were hybridized to foxd1 and foxg1a DIG-labelled antisense RNA probes. Arrows point at normal expression of nasal (A-A”) and temporal (B-B”) expression of foxg1a and foxd1, respectively. Dorsal views anterior left. Scale bar: 50 μm. (PDF) [file pgen.1008774.s023.pdf]

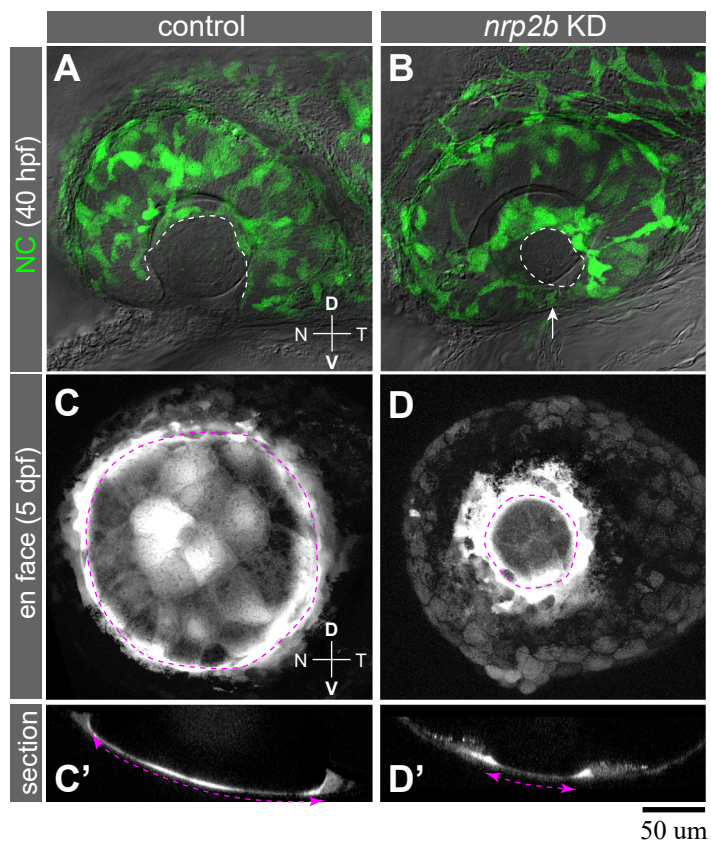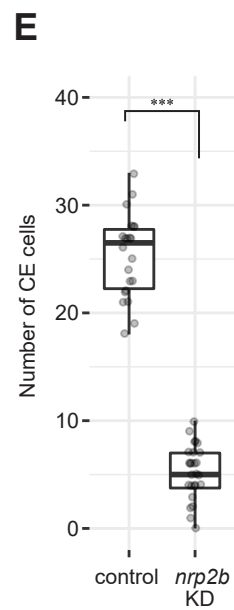

A: n=14 embryos  
 B: n=17 embryos  
 C: n= 22 embryos  
 D: n= 28 embryos

Supplement: S14 Fig — (A-B) NC cells (green) that migrated into the distal side of the eye form a NC-free area over the lens delineated by leading edge cells forming an open ring structure (stippled white line). Note that NC cells are absent in the ventral aspects of the distal eye at 40 hpf in the embryos injected with a control morpholino (A, n = 14 embryos). Upon nrp2b morpholino knock-down (KD), the NC-free area over the lens became smaller (B, stippled white circle; n = 17 embryos) with ectopic NC cells in the ventral part of the distal eye (arrow). Orientation: nasal (N), temporal (T), dorsal (D) and ventral (V). (C-D’) At 5 dpf the corneal endothelium (grey) forms a monolayer of cells over the lens, which appear surrounded by a ring of annular ligament cells (stippled magenta circle) in en face views (C-D). (C’-D’) Corresponding transverse section views along the nasal-temporal axis of a control and nrp2b-KD embryo shown in C-D. Stippled double-head arc arrows (magenta) show the extent of the corneal endothelium. Scale bar: 50 μm (E) Corneal endothelial cells of control embryos at 5 dpf are composed of 25.3 ± 3.8 cells (n = 22), whereas those of nrp2b-KD embryos are 4.8 ± 2.7 cells (n = 28). ***Welch Two sample t-test p-value<2.2 x10^-16^ (t = 20.9, df = 36.3). (PDF) [file pgen.1008774.s024.pdf]
