# Supplementary material for: Pax6 organizes the anterior eye segment by guiding two distinct neural crest waves
Source: PLoS Genet. 2020 Jun 17;16(6):e1008774. doi: 10.1371/journal.pgen.1008774 (PMC7323998; doi:10.1371/journal.pgen.1008774)
Supplement: S1 Method — (DOCX) [file pgen.1008774.s025.docx]

**Morpholinos**

***pax6a* and *pax6b***

*pax6a* and *pax6b* genes were translated from two in-frame ATG start codons [1]. The ATG regions of the two *pax6* genes share high sequence similarity. Both *pax6* genes were thus efficiently knocked-down by a cocktail of two morpholinos directed to the first and second conserved ATG region of the two *pax6* genes (S12 Figure). A p53-morpholino was co-injected at 100 µM with both *pax6a/b*- and corresponding mismatch morpholinos (each 250 µM) to suppress unspecific morpholino-mediated cell death [2]. Each embryo received less than 2 nl of injection mix. Antisense morpholinos were synthesized by Gene Tools, LLC, OR. The sequences are as follows:

1ATG_pax6ab: 5’-GTTATGGTATTCTTTTTGAGGCATT-3’;

2ATG_pax6ab: 5’- ACTGTGACTGTTTTGCATCATGGAC-3’;

1ATG_pax6ab_5-mismatch: 5’- GTTAaGcTATTCTTaTTcAcGCATT-3’;

2ATG_pax6ab_5-mismatch: 5’- ACTcTcACTcTTTTcCATCATcGAC-3’;

p53-MO: 5’-GCGCCATTGCTTTGCAAGAATTG-3’.

Small letters indicate the mutated sites in mismatch morpholinos.

**Transmission electron microscopy**

Embryos were fixed with 2.5% (w/v) glutaraldehyde, 2% (w/v) paraformaldehyde, 0.1 M PIPES (pH 7.0) overnight at 4^o^C, followed by secondary fixation for 1 h at 4^o^C in 0.8% (w/v) K_3_[Fe(CN)_6_], 0.5% (w/v) OsO_4_, 0.1 M PIPES (pH 7.0). The samples were contrasted with 2% (w/v) uranylacetate in 25% (v/v) ethanol at 4^o^C for several days. After gradual dehydration through an ethanol series (50%, 70%, 90%, 95% and 100%) and 1,2-propylene oxide, the samples were embedded into epoxy resin (glycid ether 100). Sections (70 nm) were prepared with a Leica EM UC6 ultramicrotome and recovered on Formvar-coated slot grids. Images were obtained with Zeiss T109 and LEO 910 transmission electron microscopes operated at 80 kV.

1. Nepal C, Hadzhiev Y, Previti C, Haberle V, Li N, Takahashi H, et al. Dynamic regulation of the transcription initiation landscape at single nucleotide resolution during vertebrate embryogenesis. Genome Res. 2013; doi:10.1101/gr.153692.112

2. Eisen JS, Smith JC. Controlling morpholino experiments: don’t stop making antisense. Development. 2008; doi:10.1242/dev.001115
