# Supplementary material for: Pax6 organizes the anterior eye segment by guiding two distinct neural crest waves
Source: PLoS Genet. 2020 Jun 17;16(6):e1008774. doi: 10.1371/journal.pgen.1008774 (PMC7323998; doi:10.1371/journal.pgen.1008774)
Supplement: Attachment — Submitted filename: Reviewer rebuttal final 23122019.docx [file pgen.1008774.s027.docx]

*Responses to Reviewer's Comments and Questions*

***Reviewer #1****: In this manuscript, Takamiya et al. aim to address a number of fundamental questions related to cranial neural crest and contributions to eye development. The authors use timelapse imaging and cell tracking to conclude that there are two waves of NC migration around the eye. They find that zebrafish pax6 mutants exhibit defects in anterior segment development, and NC cells appear to migrate aberrantly. pax6 mutant eyes exhibit disrupted expression of molecules known to affect cell migration, as well as defects in the lens, which is known to play an organizing role in the anterior segment.*

*1- There is a substantial amount of data in this manuscript, addressing an important and understudied aspect of eye development, however, in this form, there are concerns regarding some of the authors’ conclusions, related to a number of different key points in the manuscript. General concerns and specific points are detailed below.*

We thank this reviewer for her/his thorough evaluation of our manuscript. As these aspects of eye development were not analyzed in much detail in the zebrafish model before, it required a substantial effort to generate and validate the tools and to characterize the basic properties. As such, we were short of space to introduce and accommodate all the experiments and data. From this reviewer’s concerns and comments, we concluded that we were not explicit enough at several places. We also took the concerns of the reviewer as an indication that we should re-work the order of presentation. We re-structured the results section to introduce better the experiments and to present them in a more coherent and logical fashion. We hope this helps to understand our results as well as our language.

*2- General comments:*

*• The language is confusing with respect to descriptions of the different NC populations, cell tracking and cell migration. For example, the word “origin” is used to describe a specific position for the secondary NC, yet the cell tracks shown go further back in time, and to a similar spot as the primary NC. If the authors consider this an “origin” with respect to sox10 transgene expression, the language should be made more precise.*

Thank you for pointing this out. We are a bit puzzled by the comment that the “origin” of the two populations were the same. We modified the figure for better presentation. Clearly, the 2^o^NC derives from the medial dorsal part of the neural tube and not from the lateral regions from which the 1^o^NC migrated out earlier (new Fig. 3A’). This is also evident on the tracks shown in the supplemental S5 Video. Furthermore, we mapped the origin of the 2^o^NC by *in situ* hybridization to the dorsal region of the diencephalic mesencephalon border region (new Fig. 4). The in situ data are totally consistent with our tracking data. While 82% of cells (n=39 cells) from the domain of the 2^o^NC ended up in the distal eye, in total only 3.6% (n=112) 1^o^NC cells migrated to the distal side. There is a slight overlap at the edges of the medial (2^o^NC) domain and the lateral (1^o^NC) domains between 1^o^ and 2^o^ NC which may account for the occasional cells to end up in the other compartment. This conclusion is further supported by the observation that most of the lineages (*n* = 113; 91%) produced a NC population that exclusively consisted of either 1°NC or 2°NC cells (new S7 Figure). Thus 1°NC and 2°NC cells mostly belong to distinct lineages which is consistent with a distinct origin. Ten cells (9%) displayed a mixed lineage suggesting that 1^o^NC and 2^o^NC are not totally distinct in their potential to migrate to and differentiate into AS structures,

The two populations have also temporally distinct features. The temporal distinction is reflected in longer maintenance of *sox10* expression in 2°NC cells (Fig. 1K), strong second wave expression of the Wnt reporter in 2°NC cells (Fig. 2F) and characteristic late accumulation of 2°NC cells at the dorsal rim of the eye prior to immigration into the eye from the dorsal side as single cells hours later than 1^o^NC cells (Fig 2G).

*3- With respect to cell migration, migration “modes” and transformations are referred to, especially in the discussion (starting at line 385), and such data are not adequately presented in the manuscript to generate such conclusions; single cell behaviors are not resolvable.*

We apologize, for space considerations, we had deleted the data from the previous version of the manuscript. We added the data again as supplemental data and briefly refer to these figures and the differences in the results section (Page 5). A seen in TEM (S1 Figure D-F) 1^o^NC migrate as a sheet of highly connected cells. In contrast 2^o^NC accumulate at the dorsal rim of the eye cup (Fig 1G) and then invade the distal eye as single cells (Fig 2G) with mesenchymal morphology (Fig 1I) and highly distal-oriented movement (S6 Figure) significantly different than the 1^o^NC.

*4- • A direct correlation between sox10 transgene-positive cells and cornealEndo transgene-positive cells is missing: it is unclear how many of the AS cells are actually primary vs secondary. If the authors want to draw conclusions in which NC waves are connected to the corneal endothelium, this is an important point. I understand that there is a technical issue in which the authors may not have the appropriate tool in hand to do bona fide lineage tracing; in this case, caution should be exercised with respect to the conclusions that can be drawn.*

We agree with the reviewer that a limitation is that the expression of the *sox10* locus is turned off with the migration of the 2^o^NC to the eye. However, the translated GFP and EOSFP persist at least until 48 hpf. Therefore, we can follow the immigration of the 1^o^NC into the anterior chamber unequivocally. To cover the gap between 48 hpf and expression of the endothelial marker gene at around 60 hpf we have carried out lineage tracing using the inducible CreERt2/loxP system to permanently label the cells. To visualize AS structures without being intervened by pigment, we used the Tg(ubi:Zebrabow-Sa132) (Albert Pan et al. 2013) LoxP reporter line established in pigment-free Casper mutants, mitfaw2/w2;mpv17a9/a9 (White et al. 2008). LoxP reporter embryos at one-cell stage were injected with a Tol2 transposon plasmid DNA encoding sox10:CreERT2, with Tol2 transposase mRNA for transient expression of Cre recombinase in NC cells. We used two time windows for CreERt2 activation from 2 to 28 hpf and from 22 to 28 hpf. A total of 204 cells (n=10 embryos) and 395 cells (n=40 embryos) in the eye were found to have originated from sox10-positive NC cells for 2-28 hpf and 22-28 hpf Cre activation, respectively. Consistent with our previous observations, the corneal endothelium received NC cell contribution (Table R1, below). The iris and sclera contain high numbers of NC descendants (Table R1).

|  | Tissue | | | | | | | | |  |
| --- | --- | --- | --- | --- | --- | --- | --- | --- | --- | --- |
| 4OH-TAM | corneal Endothelium | | Iris | Sclera | | Total | |  |  |  |
| 2-28 hpf | 10 (4.9%) | 132 (64.7%) | | | 62 (30.4%) | | 204 | |  |  |
| 22-28 hpf | 29 (7.3%) | 278 (70.4%) | | | 88 (22.3%) | | 395 | |  |  |

Table R1 Fate analysis of NC cells.

Tg(zebrabow);casper embryos were injected with sox10:creERt2 expression construct at the one-cell stage. CreERt2 recombinase was activated by applying 1 µM 4-OH tamoxifen during either 2-28 hpf or 22-28 hpf. Embryos were imaged at 28 hpf and 5 dpf for pairwise comparison of identical individuals.

Fig R1 sox10-positive NC cells contribute to the AS structures: A) NC cells at 28 hpf are shown in yellow. Blue color indicates default expression of Zebrabow transgene (RFP). In the distal side of the eye several NC cells are labeled. A’) Transverse cross section at the level of the lens shows NC cells between the lens primordium and surface epithelium. B) The same eye shown in A was examined at 5 dpf for the NC contribution to AS structures. Ir: iris, Ln: lens, AL: annular ligament: Sc: sclera. Orientation of the embryo is shown, dorsal (D) up and rostral (R) left. Asterisks are recombination events derived from ectopic Cre driven under 7.2 kb sox10 regulatory element. (C-C’) Tg(sox10:mem-tdEosFP) line shows weak ectopic activation of reporter in the lens primordium at 18 hpf. Transverse section is shown in C’.

The absolute NC contribution to the various eye structures is, however, difficult to assess from these data as differential cell divisions may have distorted the picture at 5 dpf. Moreover, we had hoped that we may be able to discriminate 2^o^NC cells by applying tamoxifen at the two distinct time windows. Both treatment regimes lead to labeling of sclera cells. The 22-28 hpf treatment yielded slightly less labeled sclera cells and slightly more corneal endothelial cells (Table R1). However, this result (if significant at all) is by far not as black and white as our real-time analysis. The reason is that this approach is flawed by the stability of the CreERt2 recombinase protein that is still present in the 1^o^NC cells after *sox10* expression has been silenced. We most likely induce still recombination in 1^o^NC cells already located in the proximal eye. As such, we cannot discriminate between the cell populations. Long-term labeling of 2^o^NC requires not only temporal but also spatial control of CreERt2 activation. Despite the mosaicism of expression inherent to these DNA injections, when we examined embryos at 28 hpf, we noted many labelled NC cells making tracking very difficult over subsequent stages. Moreover, we cannot exclude that zebrabow expression becomes detectable after 28 hpf. Thus real-time lineage tracking would be required which is a problem over the required long time span.

Altogether to answer this question of the reviewer satisfactorily is an entire new study. There is ample prior evidence of neural crest contribution to the corneal endothelium. Multiple lines of provided here point out that also in fish the NC contributes to corneal endothelium formation. We thus question whether the effort to close this 12 hours gap between loss of *sox10* driven expression and appearance of endothelial marker *Tg(corneaEndo:GFP)* is justified.

*5- • In the pax6 mutants, the authors examine the expression of some signaling pathway components known to affect cell motility and guidance. The authors conclude that a function of pax6 is in guidance of neural crest migration, yet no direct test is carried out. If the authors are hypothesizing that changes in the expression of these guidance molecules are affecting neural crest migration, the authors could manipulate some of these genes (via mutants or morpholinos) to directly test a role in guiding primary or secondary neural crest migration.*

We conducted antisense morpholino-mediated knockdown of guidance molecules (new Fig 9) *sema3aa*, *efnb2a* and *nrp2b*, which are expressed in the lens, the lens/retina, and NC cells, respectively. No significant effects on NC migration and AS formation were observed upon knockdowns of *sema3aa* and *efnb2a* (not shown). Redundancies with related genes, inefficiency of the morpholino could account for these non-conclusive results in addition to a lack of function in NC cell guidance in these two cases.

Abnormal NC migration and reduced corneal endothelial cells were observed when we knocked-down *nrp2b* (Fig. R2). The 1°NC cells of *nrp2b*-knockdown embryos migrated far into the distal half of the eye, phenocopying the behavior of 1^o^NC cells in *pax6b-/-;pax6a-/-* mutants (Fig. R2B, arrow). In contrast to the double mutant, however, 2°NC cells in *nrp2b*-knockdown embryos migrated normally into the anterior segment of the eye. Thus, altered *nrp2b* expression in NC cells of the mutants is only one aspect of the defects causing abnormal 1^o^ and 2^o^NC migration. It also suggests that the two populations use different guidance cues, - in total agreement with our other data pointing at the 2^o^NC cell group as a NC population distinct from the 1^o^NC.

We counted less endothelial cells in the knock-down (Fig. R2E). However, this may just be a reflection of the smaller eye. The presence of the corneal endothelial layer is also consistent with the observation that 2^o^NC still move correctly into the eye in the nrp2b knockdown. We added these data as S14 Figure.

Fig. R2 Nrp2b is required for correct guidance of NC cells and eye development.

(A-B) NC cells (green) that migrated into the distal side of the eye form a NC-free area over the lens delineated by leading edge cells forming an open ring structure (stippled white line). Note that NC cells are absent in the ventral aspects of the distal eye at 40 hpf in the embryo injected with a control morpholino (A, *n*=14 embryos). Upon *nrp2b* morpholino knock-down (KD), the NC-free area over the lens became smaller (B, stippled white circle; *n*=17 embryos) with ectopic NC cells in the ventral part of the distal eye (arrow). Orientation: nasal (N), temporal (T), dorsal (D) and ventral (V). (C-D’) At 5 dpf the corneal endothelium (gray) forms a monolayer of cells over the lens, which appear surrounded by a ring of annular ligament cells (stippled magenta circle) in “en face” views (C-D). (C’-D’) Corresponding transverse section views along the nasal-temporal axis of a control and *nrp2b*-KD embryo shown in C-D. Stippled double-head arc arrows (magenta) show the extent of the corneal endothelium. Scale bar: 50 µm. (E) Corneal endothelial cells of control embryos at 5 dpf are composed of 25.3 ± 3.8 cells (*n* = 22), whereas those of *nrp2b*-KD embryos are 4.8 ± 2.7 cells (*n* = 28). ***Welch Two sample *t*-test *p*-value<2.2 x10^-16^ (*t*=20.9, *df*=36.3).

*6- • In general, the manuscript is a bit unfocused and difficult to read. For example, how exactly does wnt signaling actually fit into this? The experiments presented suggest that it does not play a role in AS development.*

Wnt signaling has previously been shown to be a crucial component of neural crest specification in posterior neural crest redefined here as 1^o^NC (Raible 2006). We thus **had to** test the involvement of Wnt signalling in eye 2^o^NC specification. As a first assay, we analyzed the Wnt reporter and found that it is highly activated late in 2^o^NC in a second wave of expression relative to its initial activation in 1^o^NC cells (current Fig 2). We then tested the potential role of Wnt signaling in 2^o^NC specification by treatment with a Wnt signaling inhibitor. We did not find evidence for a role in corneal endothelial development (current S3 Figure E-H). We found, however, a moderate reduction of the number of 2^o^NC cells prior to immigration into the eye (current S3 Figure D).

Given its role as powerful marker for 2^o^NC and the previous published function in 1^o^NC specification (Dorsky, Moon, and Raible 1998), we believe it is necessary to include the data on Wnt signaling. We checked the relevant passages in the results section (page 7) where we introduced the experiments with the Wnt reporter to make sure that the reader is adequately informed. We also arranged the results section introducing Figures and descriptions in the text in the right order. We believe that this is part of the reviewer’s problems. We hope that we have solved the issue to his/her satisfaction in this way.

*7- Another example is Figure 7 (*current Fig 10*), in which the TGF-beta signaling pathway is examined, yet this is unconnected to the rest of the manuscript. Can TGF-beta be connected to the rest of the manuscript by testing it in the context of pax6 mutants, or adding it back to rescue pax6 mutants?*

There is evidence that NC derived stem cells can be directed towards endothelial development by exposure to TGF-beta signals (John et al. 2011). Moreover, TGFbeta2 has been implicated in AS segment development in mice (Saika et al. 2001)(Ittner et al. 2005). So there was some indirect previous evidence suggesting an involvement. It was thus important to check whether TGFbeta signaling plays a role in the control of 2^o^NC. We added statements of motivation in the results section.

The reviewer indicates that there is no connection to the Pax6 mutants. Considering that 1. blocking TGFbeta abolishes endothelium formation (Fig. 10 O-P, S10 Movie), 2. the lens is a source of TGFbeta signals (Ittner et al. 2005; Saika et al. 2001)(Fig 10A-E) and 3. the lens is missing in Pax6a/b mutants (Fig. 6) there is a clear link between the Pax6 mutant data and TGFbeta signaling.

We have followed the suggestions of the reviewer and embarked on the rescue of the *pax6a/b* mutant phenotype by implantation of TGFbeta2-soaked beads into the mutant eye (Fig. R3). We observed an increase of corneaEndo:GFP cells in the *pax6a/b* deficient embryo implanted with the bead soaked in human TGFbeta2 (20 µg/ml; Fig R3B) relative to the implantation control (BSA soaked beads, Fig. R3A). These GFP-positive cells also line the inner surface of the cornea (Fig. R3B’) closing the gap seen in pax6ab mutant (Fig 6K-K’ In the paper) and the implantation control (Fig.R3A’). However, when one compares the morphology of the rescued cells (Fig. R2B’) with those of the wildtype control (Fig. R3C’), it is evident that the GFP positive cells in the mutant with implanted TGFbeta2 bead are not as flat and large as those in the wildtype control. Thus the rescue does not seem to be complete. This is also reflected in the overall malformed structure of the eye.

Fig. R3 Implantation of TGFbeta soaked beads partially rescued the pax6a/b mutant phenotype

A-C: Pax6a/b was eliminated by injecting a Pax6a morpholino into *pax6b^-/-^*; *Tg(corneaEndo:GFP)* embryos (A, B). An agarose bead soaked with BSA (A) or human TGFbeta2 (B) was implanted into these *pax6a/b* deficient embryos. Unoperated wild type *Tg(corneaEndo:GFP)* control is shown in C. Overlays of the GFP fluorescence as well as the total reflection images from the mutant (A, B) and the unoperated wildtype control Tg(corneaEndo:GFP) embryo (C) are shown. In comparison to the BSA-bead control (A), a much higher number of GFP-positive cells is seen in the mutant eye implanted with a TGFbeta2 soaked bead. These cells resemble at first glance corneal endothelial cells in en enface view (B). However, in cross sections (B’), it is obvious that these cells are not totally differentiated into the characteristic flat cells lining the interior surface of the cornea at this stage in *pax6a/b* wildtype (C’) transgenic embryos. Thus, all together implantation of a TGFbeta2 bead increased the number of corneaEndo:GFP expressing cells. However the rescue was partial.

These preliminary data suggest that indeed TGFbeta2 signaling can rescue the effect partially at least. These observations are in line with the observed requirement of TGFbeta2 signaling in the differentiation of the endothelium in wildtype embryos. These data are also consistent with the role of the lens as a source of TGFbeta2 and that lack of the lens will lead to impaired TGFbeta2 signalling thus affecting AS differentiation in the *pax6a/b* mutants. This facts being consistent with a necessary role of TGFbeta2 in AS differentiation, we never claimed, however, that TGFbeta signalling were sufficient to structure the anterior segment and replace pax6a/b function. The most likely explanation is that other factors play a role too. Our finding of an involvement of Nrp2b heralds such other processes independent of TGFbeta2 signaling that are affected in the mutants.

These implantation experiments are extremely difficult to carry out. In addition, these experiments are prone to artefacts. The surgery or even the presence of an inert piece of agarose as a lens substitute causes uncontrollable interference with AS restoration. Another problem is to get the dose of TGFbeta2 right. We cannot exclude teratogenic effects by too high and too long exposure. Our TGFbeta signaling reporter assay as well as the TGFbeta2 mRNA expression suggested that TGFbeta signaling in periocular mesenchymal cells is temporally limited (Fig.10M). Thus, we believe that to continue this type of experiments will not lead to the expected solid outcome that the reviewer hopes for.

*Specific points:*

*8- • Figures 1 and 2 (*current Fig 1 and 3*): the authors use high resolution imaging and cell tracking to examine neural crest migration around the eye. They conclude that there are two waves of NC migration, with different origins and destinations. The difference in “origins” is confusing, as the tracks in Figure 2* (current Fig 3) *appear to extend back to a start position similar to the primary cells. The difference in destinations is clear at 31 hpf, yet both populations may contribute to the corneal endothelium shown in Figure 1J-L’* (current Fig 5). *Can the authors comment on their confidence in the distinctions between these origins and endpoints? Is there a lineage tracing tool that can be used (sox10:Cre combined with a zebrabow type reporter) to identify the endpoints and contributions more definitively?*

We are absolutely confident that the origins and the destinations are different between the 1^o^ and 2^o^NC. We provide cell counts from tracking individual cells. We found that: “The lineage tree analysis on manually corrected data from an embryo identified comparable lineage sizes for 1° and 2°NC of 57 and 46 lineages, respectively (Fig 3F; S4-5 Video; S6 Figure). Most of the lineages (*n* = 103 of 113; 91%) produced a homogeneous NC population that exclusively consisted of either 1°NC or 2°NC cells, showing that 1°NC and 2°NC cells mostly belong to distinct lineages. Ten cells (9%) displayed a mixed lineage suggesting that 1^o^NC and 2^o^NC are not totally distinct in their potential to migrate to and differentiate into AS structures, The tracking analysis furthermore confirmed the predominant migration of 2°NC cells into the distal side (*n* = 32 of 39 NC cells; 82%), which was further supported by local photoconversion and time lapse analysis (Fig 3G-H; S6 Video). In contrast, 1°NC cells rarely ended up in the distal part of the eye (*n* = 4 of 112 cells, 3.6%). Thus, the two distinct NC cell groups contribute predominantly to distinct regions of the eye i.e. the proximal and distal regions, respectively.

We changed the color scheme of the tracks in Fig 3A’, 3B-C to make clear that the origin of the tracks of 1^o^ and 2^o^NC is definitely different. Tracks of 1^o^NC cells are exclusively derived from the lateral earlier migrating neural crest. In contrast, 2^o^NC are derived predominantly from the dorsomedial region at the diencephalic mesencephalic boundary. This location of the origin was independently mapped by in situ hybridization with various probes specific to this region and the NC cells (Fig.4). In situ hybridization took advantage of the later onset of the sox10 expression which was turned off from the laterally located earlier migrating 1^o^NC cells earlier. For our response to the request of independently tracking see also our response to point 4 of this reviewer.

*9- • Figure 2* (current Fig 3) *and S3* (current S4): *the forward tracking of the primary NC (2D-D’ and S3D-D’) appear somewhat different from each other in the two different embryos. In the second embryo, are the cells all converging toward a specific spot behind the eye?*

This appears only like this as it is not only the cells that move but also the optic cup; it increases its volume with its temporal border (arrowheads, Fig R4) extending toward the posterior end during development. The eyes effectively move backwards relative to the nasal border of the optic cup (Fig. R4). This posterior movement is part of optic cup morphogenesis (Kwan et al. 2012). As a result, NC cells on the proximal surface of the optic cup appear to “migrate” backwards.

Fig. R4: Backward eye movement after 17 hpf.

Confocal time lapse images showing NC cells (green) merged on bright field view. The embryo was embedded in 0.5% low melting point agarose. A sequence of images from 17.5 to 27.5 hpf is shown. The position of the temporal border at 17.5 hpf is shown with a white vertical line. The temporal border of the optic cup (arrowheads) is moving in posterior direction as the developmental stage proceeds. Orientation: posterior (right).

*10- • Figure 1 and 2 (*current Fig 3*) and conclusions summarized in the Discussion (lines 385-395): the authors use their imaging to describe some of the migration behaviors of the primary and secondary NC populations. These behaviors are difficult to see, since single cell morphologies are not really resolvable; unmerged Eos images would be helpful to include to assess migration patterns and behaviors.*

Thanks. The phrasing of this section was influenced by a previous version where these data were included. We have added data to the supplement to support our claims (S6 Figure). We also systematically measured and documented the migration direction of a large number of 1^o^ and 2^o^ NC cells (Fig. R5).

Fig. R5 Migration angle of 1^o^NC and 2°NC cells, when 2^o^NC clusters at the dorsal edges of the eyes dissolve (22 hpf), are different. (A, B) Movement angle histogram for NC cells destined for proximal (A, 1^o^NC) and distal (B, 2^o^NC) sides of the eye. Movement trajectories of an individual NC cell were projected on an horizontal plane and the angle relative to the anteroposterior axis was measured every 3 min 24 s over 10 time points from 44 and 37 cell trajectories, respectively. Each bin is 10°. Black arrows show median direction (56.5° and 83.6° for proximal and distal selection, respectively). Wallraff test of angular distances between two groups showed significant difference, *p*-value < 2.2x10^-16^. (C, D) Centered tracks starting from 22 hpf for proximal (C, 1^o^NC) and distal (D, 2^o^NC) destined NC cells. The starting position is set to 0 (bottom center).

*11- The Discussion contains some broad statements about transformations of collective cell behavior, “mesenchymal” cell shapes, and use of the word “misrouting”; the data presented in this manuscript are not adequate to support these conclusions. It would be helpful to quantify these phenotypes at the single cell level.*

We added additional data to support our case of different ways of migration of the two NC populations in wildtype and pax6 mutant embryos (S9 Video). See response to point 10.

Fig. R6: In pax6b-/-;pax6a-/- double mutants (right panels; *n*=3 embryos), the 2°NC cells (green) do not migrate into the distal side of the eye when wildtype and wildtype-like embryos do (left panels). In double mutants, 2°NC cells did not dissolve into single cells unlike wildtype-like embryos and its cluster persists at the dorsal edge of the eye. A selected image from S9 Video is shown. 1°NC cells are shown in magenta. 1^o^ and 2^o^ NC cells were visualized by conversion of green EosFP to magenta. 2^o^NC cells recovered green whereas 1NC cells did not and remained magenta.

*12- • Figure 1J-L* (current Fig 5): *the authors use the corneaEndo:GFP transgenic line. More validation is necessary to demonstrate that this is corneal endothelium: in Figures 1L’-L’’, a small arrow is placed to mark the epithelium, but what this arrow points to is difficult to see. In addition, it is not clear exactly how the cells marked by this transgene are related to the sox10 transgene positive cells.*

We added additional panels to Fig.5C’’ (see also below). Overlay of total reflection images and the fluorescence images anchor the fluorescence to specific structural features. These data clearly show that the transgene is expressed in the inner endothelial layer of the cornea.

*13- • Figure 1N-O (*current S10 Figure*): the sox10 mutant cornea phenotype is difficult to interpret. The authors state that the endothelial layer is missing. Can this be quantified? For example, is the number of corneal endothelial cells different? Or the thickness of the corneal endothelium? Can the authors zoom out to show the phenotype in a broader view?*

We have analyzed two wildtype-like siblings and four mutant embryos by EM. The endothelial layer that forms a monolayer of flat cell in wildtype has not formed in each of the mutants. Instead, we found at the inner surface of the cornea an abnormal endothelial layer in all mutants (Fig. R7D to F) but not the wildtype sibling corneas (Fig. R7A to C).

Fig. R7: Low magnification TEM overview of the corneal endothelium from Sox10 t3/t3 mutants (D-F) and their wildtype-like siblings (A-C). The corneal endothelium is pointed by arrows in siblings (A-C). (D-F) sox10 t3/t3 mutants show variety of abnormal phenotypes of the corneal endothelium, including edema formation (asterisk, D), thinning of the endothelium layer (arrowhead, D), vacuolated cells with irregularly thick endothelium layer (arrowheads, E-F).

We have employed a morpholino approach to assess the number of corneaEndo:GFP cells in sox10 deficient embryos (Fig. R8). We found a significant reduction of GFP-positive cells in *sox10* morpholino injected embryos versus control embryos (Fig. R8). Moreover, the cells appeared thicker and less regular in shape than the GFP-positive cells in control embryos (Compare Fig. R8A’ and R8B’) in agreement with the irregular shape of these cells seen in the electron micrograph.

Fig. R8 Knock-down of Sox10 causes an eye phenotype

(A-B’) At 5 dpf the corneal endothelium (gray) forms a monolayer of cells over the lens, which is surrounded by a ring of annular ligament cells (stippled magenta circle) in en face views (A-B). (A’-B’) Corresponding transverse section views along the nasal-temporal axis of a control and *sox10*-KD embryo shown in A-B. (A’-B’) Stippled double-head arc arrows (magenta) show the extent of the corneal endothelium. Note in agreement with the electron micrographs the endothelial cells appear thicker and more irregular than the control endothelial cells in A’. Orientation: nasal (N), temporal (T), dorsal (D) and ventral (V). Scale bar: 50 µm. (C) Control embryos at 5 dpf have 28.6 ± 3.6 corneal endothelial cells (*n*=25), whereas *sox10*-KD embryos show 12.8 ± 6.7 cells (*n*=28). ***Welch Two sample *t*-test *p*-value=1.3 x10^-13^ (*t*=10.7, *df*=42.1).

*14- • Figure S6* (current S3 Figure): *the implications of the wnt inhibitor experiments are unclear. Treatment with IWR-1 does affect secondary neural crest cells, but not formation of the corneal endothelium. Does this mean that the secondary neural crest cells are not important for the corneal endothelium?*

No, our data do not support the conclusion of the reviewer. Rather our data suggest that **wnt signaling** is not important for 2^O^NC specification and the formation of the endothelium of the cornea. We found a reduction in 2^o^NC cells when embryos were treated with Wnt inhibitor. However, this did not abolish the formation of the 2^o^NC entirely. We interpret our data that the embryo can compensate for the reduction of 2^o^NC cells in Wnt inhibitor treated embryos during subsequent development.

*15- • Figure 4A-B (*current Fig 1KL*): the authors use tdEos photoconversion to examine when the sox10 transgene is turned off. Strictly speaking, this experiment examines when translation of tdEos stops, not transcription; the title of the chart in 4B should be edited.*

Yes, this is correct. Thanks for pointing this mistake out. In most cases transcription is tightly coupled to translation. Moreover, we have verified our data also by *in situ* hybridization to *sox10* mRNA (Fig 4A). We thus, believe that our conclusion is valid but of course we should state throughout “expression”. We edited the title of the chart and state now: “*de novo* sox10 expression “

*16- • Table 1 and Figure 6* (current Fig 7): *the authors argue that Pax6a/b dose correlates with NC migration and anterior segment defects. Neither dosage nor phenotypes were quantified beyond numbers of wild type or mutant alleles, or in the case of phenotypes, numbers of embryos. Given recent studies of transcriptional compensation in mutant backgrounds, if dosage is an crucial point, it would be important to know quantitatively how pax6 expression and function might be affected in each genotype (for example, using qRT-PCR to determine level of expression, or luciferase assays to determine function of the missense mutant). In terms of phenotypes, was the number of cells exhibiting migration defects affected? Or the just the numbers of embryos?*

We followed here entirely the murine concepts of *Pax6* dose being correlated with the severity of the phenotype (Kroeber et al. 2010; Schedl et al. 1996). Indeed, we see a similar correlation. We thus believe it is not necessary to reanalysis this issue further in detail than summarized in Table 1. We see significant difference between gene numbers and 2°NC cell migration defects (see Table 1). 1^o^NC cells migrate aberrantly to the distal side of the eye only in the double mutant, which correlates with loss of the lens. The reviewer is entirely right we can not state “dose of Pax6”. We thus carefully went through the section and the rest of the ms to replace dose of pax6 with number of pax6 genes.

*17- • Figure 4 (*current Fig 8*): the authors use in situ hybridization of a number of different eye genes to conclude that patterning is not affected in the pax6 mutants. The data (specifically the genes assayed) suggest that dorsoventral and proximodistal patterning are not affected, but the statement that patterning is “unaffected” is a bit of an overreach in the absence of nasotemporal markers (such as foxg1 and foxd1).*

We carried out in situ hybridization experiments with foxg1 and foxd1 antisense RNA (Fig R9). We found that expression of these markers of nasotemporal polarity are not affected in *pax6b-/-* single and *pax6b-/-;pax6a-/-* double mutants. We included these results in S13 Figure. To avoid further confusion, we removed the words “independently of optic cup patterning” from our title and now it reads “Pax6 organizes the anterior eye segment by guiding two distinct neural crest waves”.

Fig. R9 Nasotemporal polarity of the eye is not affected in pax6a/b double mutants.

Embryos (28 hpf) were hybridized to foxd1 and foxg1a dig-labelled antisense RNA probes. Dorsal views anterior left. Arrows point at normal expression of nasal (A-A’’) and temporal (B-B’’) expression of foxg1a and foxd1, respectively.

*18- • Figure 4* (current Fig 7): *the authors find alterations to cell guidance molecules in the pax6 mutants, and a major conclusion is that pax6 guides the neural crest. The data do not necessarily support a role for guidance: If the authors hypothesize that some of these molecules are important for NC guidance, expression of these molecules should be directly manipulated in a wild type background (via genetic mutation or morpholino oligonucleotide) and NC migration examined.*

We followed the reviewer’s suggestion. See comment to response #5 (Fig. R2) for the description of the results.

*19- • Figure 7: the TGF-beta-related experiments in this figure seem unrelated to the rest of the manuscript. Because the lens transplant seemed to rescue some of the AS phenotypes in Figure 6, can the authors use a TGF-beta soaked bead to see if TGF-beta is sufficient to rescue the phenotype? Or examine sox10 and pitx2 expression in embryos treated with the TGF-beta inhibitor?*

Please see comment #7 and Fig. R3 above for a comprehensive response to this concern. We have analysed *sox10* expression in inhibitor treated embryos. We did not observe prolongation of sox10 expression in TGFβ inhibitor-treated embryos in contrast to the effects seen on mammalian NC cells in vitro (John et al. 2011).

*20- • Discussion: there are statements that appear to be slightly over-reaching, based on the data presented in the manuscript. These include language regarding cell behavior and “transformations” thereof (lines 385-395); this is not directly shown or addressed in this manuscript, as the transgenic lines do not provide enough cellular resolution to make these conclusions. The word “misrouted” (line 395) is strong and suggests guidance to a different location, which is unclear.*

We appreciate the comment of the reviewer as it implies that we have not stated our case clearly enough. We have addressed these issues raised already in the comments to the results section several times in our responses #2, #3, #10, #11, and #18. As mentioned in details in these sections above, we have rearranged the paper and added more data to strengthen our view (see Fig 1G, I; Fig 2G, S1Figure D-F; S6 Figure; Video S9). Given the fact that we barely detect 1^o^NC cells in the distal segment in wt embryos, we believe that the term misrouted is justified for the observation that 1^o^NC cells end up in the distal segment in the double mutants*.*

*Minor points:*

*21- • Figure 3* (current Fig 4): *the term DMB should be defined*

Thank you. We added the explanation to the legend.

*22- • Line 325-326: “Lens-derived TGF-beta2 organizes periocular mesenchyme differentiation to form the AS in mice.” This statement needs a citation.*

Yes, indeed. Thank you. We added the reference that was accidentally lost.

*Reviewer #2: This manuscript [PGENETICS-D-19-01379] by Takamiya et al. details a major research advance in our understanding of neural crest cells in the vicinity of the developing eye. The researchers demonstrate that there are indeed two separable populations of neural crest cells in this region. Furthermore, they demonstrate the key roles of Pax6 and Tgf-b in this process. Overall, this is a fascinating story and has widespread implications for the analysis of anterior segment development and the studies of anterior segment dysgenesis. Although the vast majority of experiments are well controlled and convincing, there are a few outstanding issues that remain (all outlined below):*

*Major:*

*23- 1. The examination of patterning and guidance molecule gene expression seems rather confusing. It is quite obvious that expression of guidance molecule genes is strongly affected. The conclusion, though, that patterning is unaffected seems unsupported by the data. Tbx5, aldh1a2, and vax2 show obvious, albeit mild, changes to gene expression. Yet the authors conclude that patterning is unaffected by pax6. I suggest that a quantitative approach would be required to show that such patterning genes are unaffected (qPCR of retinal tissue and/or measurement of area of gene expression). If indeed such genes are affected, the authors will need to rework their conclusions and their title.*

Thank you for pointing this issue out to us. We see it as an indication that we have not been precise enough in our statements. Previously in mice, it has been suggested that Pax6 confers positional information to the eye defining the different axes. However, our data do not support this. We clearly see that the overall coordinates of the eye have formed even in the double mutant. To assess this we relied on early expressed genes encoding mostly transcription factor genes whose expression is restricted along the various axes of the nascent eye. Of course the individual markers may be slightly affected by the changes in size and morphology of the mutant eye. We have rephrased the conclusions to make our point clearer.

From these observations, we hypothesized that guidance cues may be affected in the mutants and indeed we see differences in the in situ expression of the genes encoding these guidance cues. We have attempted to quantify by qRT-PCR as well as RNASEQ. Since the genes are all expressed in other regions of the head and collection of sufficient tissue is extremely difficult – especially as we have to consider 20 to 30 hpf embryos. The results using the whole embryo were not conclusive. We focused instead our analysis on functional experiments knocking-down the guidance molecules and assessing whether NC cells would be misrouted. Please see response to major concern 2 of this reviewer (our response #24) and also response 5 and Fig. R2).

*24- 2. A functional correlation between guidance molecule function and neural crest migration has not been established. To establish this, the authors are encouraged to perform either rescue experiments (Tg[eye promoter::guidance gene] to rescue phenotype of mutants) or knockdown (or pharmacological block) to inactivate cues.*

We have tested *sema3aa*, *efnb2a* and *nrp2b*, which are expressed in the lens, the lens/retina, and NC cells, respectively, by antisense morpholino knock-down (see response 5 and Fig. R2 of this rebuttal letter for experimental details and results). Indeed, we observed a phenotype partially resembling the pax6 mutant phenotype, in *nrpb2* knock-down embryos suggesting that nrpb2 is involved in the pathomechanism underlying ASD in pax6a/b mutants. We did not see effects with the other two morpholinos but this could have a lot of trivial reasons such as redundancies besides lack of involvement in the process.

*25- 3. The analysis of tgfb function seems somewhat incomplete. The authors have not analyzed a neural crest reporter in such experiments and this is imperative. The authors should also determine the critical period of Tgf-b function by performing treatments at differing stages.*

We have carried out treatment with the TGFbeta inhibitor to test an effect of TGFbeta signaling on NC migration. We did not detect an effect on NC migration. However differentiation of NC cells into *corneaEndo:GFP* expressing cells was strongly affected (Fig 10 OP; S10 Movie in the paper; see also Fig. R10 in this rebuttal letter below). We also did not observe prolongation of sox10 expression in TGFbeta inhibitor-treated embryos in contrast to the effects seen on NC cells in vitro (John et al. 2011). We inserted a sentence into the results section. We believe that TGFbeta is involved in later differentiation of the NC cells once these cells have entered the eye.

Fig R10 TGFbeta inhibitor (SB-505124) reduces corneaendo:GFP expression strongly.

Embryos carrying the Tg(corneaEndo:GFP) transgene marking the corneal endothelial layer were treated from 46 hpf to 87 hpf with the TGFbeta inhibitor SB-505124 (30 µM). The Upper row shows four eyes of solvent control embryos. The bottom row shows eyes of treated embryos. The expression of the corneal endothelial marker (green) is essentially abolished with strongly reduced pupil size.

As the reviewer felt the analysis being incomplete we added more data and explanation.

*Minor:*

1. *The eye has changed size in Fig 7 (*current Fig 10*), which prevents the reader from seeing periocular gene expression in the later stages. The authors should increase the size of the pictures such that the entire eye is shown.*

We replaced the pictures so that the whole eye is shown in the panel (Fig 10; Fig R10).

Fig R10 in situ hybridization analysis of tgfb2 and gfp from Tg(smad3:gfp) line.

2. *The genetic background has recently been shown to play a considerable role in phenotypic penetrance. Although it is possible that this was missed in the text, I didn’t see a mention of the genetic background for the experiments performed (AB, TB, or other). This should be included. Also, did the researchers perform any experiments on other backgrounds?*

Thank you for pointing this error out. We included the genetic background “ABO” in the materials and methods section.

**References**

Albert Pan, Y. et al. 2013. “Zebrabow: Multispectral Cell Labeling for Cell Tracing and Lineage Analysis in Zebrafish.” *Development (Cambridge)*.

Dorsky, Richard I., Randall T. Moon, and David W. Raible. 1998. “Control of Neural Crest Cell Fate by the Wnt Signalling Pathway.” *Nature*.

Ittner, Lars M. et al. 2005. “Compound Developmental Eye Disorders Following Inactivation of TGFβ Signaling in Neural-Crest Stem Cells.” *Journal of Biology*.

John, Nessy, Paolo Cinelli, Michael Wegner, and Lukas Sommer. 2011. “Transforming Growth Factor β-Mediated Sox10 Suppression Controls Mesenchymal Progenitor Generation in Neural Crest Stem Cells.” *Stem Cells*.

Kroeber, Markus et al. 2010. “Reduced Expression of Pax6 in Lens and Cornea of Mutant Mice Leads to Failure of Chamber Angle Development and Juvenile Glaucoma.” *Human Molecular Genetics*.

Kwan, Kristen M. et al. 2012. “A Complex Choreography of Cell Movements Shapes the Vertebrate Eye.” *Development*.

Raible, David W. 2006. “Development of the Neural Crest: Achieving Specificity in Regulatory Pathways.” *Current Opinion in Cell Biology*.

Saika, Shizuya et al. 2001. “Tgfβ2 in Corneal Morphogenesis during Mouse Embryonic Development.” *Developmental Biology*.

Schedl, Andreas et al. 1996. “Influence of PAX6 Gene Dosage on Development: Overexpression Causes Severe Eye Abnormalities.” *Cell*.

White, Richard Mark et al. 2008. “Transparent Adult Zebrafish as a Tool for In Vivo Transplantation Analysis.” *Cell Stem Cell*.
