# Supplementary material for: Pax6 organizes the anterior eye segment by guiding two distinct neural crest waves
Source: PLoS Genet. 2020 Jun 17;16(6):e1008774. doi: 10.1371/journal.pgen.1008774 (PMC7323998; doi:10.1371/journal.pgen.1008774)
Supplement: Attachment — Submitted filename: Rebuttal_PGENETICS-D-19-01379R1.docx [file pgen.1008774.s028.docx]

**Response to reviewers’ comments:**

*Reviewer #1: The authors have made a significant effort to answer the questions and comments posed. The reorganization of the data, and the edits to the figures are helpful, as is the thorough point-by-point response.

I have a few further questions and comments:

• Regarding the new TGFbeta2 data included in the reviewer response: I think the partial rescue is actually quite exciting, and could be included as supplementary data. I don’t think that anyone would argue that TGFbeta2 is the only relevant factor coming from the lens; the data demonstrate that TGFbeta2 is a piece of the puzzle downstream of pax6a/b and the lens.*

Thank you for sharing our excitement on partial rescue by implanted TGFbeta2 beads. We managed the successful establishment of the implantation technique and have shown the first highly encouraging data supporting the general conclusion of the present paper. We would like to develop an independent manuscript to describe the detailed analysis of these effects of the TGFb mediated partial rescue, including different TGFbeta2 doses, a comprehensive study of gene expression changes in the anterior segment to assess which structures have been rescued. We are hesitant to include our preliminary data into the Supplement at this level of analysis. Our conclusions would have to be either very restricted or would be misleading. Considering well-known adverse effects of TGFbeta1 overdoses on the corneal endothelium in a transgenic mouse model (Reneker et al. 2010) this study requires carefully controlled conditions that we cannot provide with this limited analysis. We would thus prefer to not include the data.

*• Inclusion of cell movement data is helpful (S6 Figure), however, this still doesn’t really answer the question of cell morphology. In the previous review (points 10 and 11), I had concerns regarding the use of terms related to descriptions of cell behavior (e.g. “collective behavior”, “mesenchymal cell shapes”). S6 Figure appropriately addresses directionality and processivity of movement. Extra movies are helpful (e.g. S9 Video), but without more single cell morphology information, collective cell behavior is still somewhat hard to see.*

Points 10: *10- • Figure 1 and 2 (*current Fig 3*) and conclusions summarized in the Discussion (lines 385-395): the authors use their imaging to describe some of the migration behaviors of the primary and secondary NC populations. These behaviors are difficult to see, since single cell morphologies are not really resolvable; unmerged Eos images would be helpful to include to assess migration patterns and behaviors.*

Points 11: *11- The Discussion contains some broad statements about transformations of collective cell behavior, “mesenchymal” cell shapes, and use of the word “misrouting”; the data presented in this manuscript are not adequate to support these conclusions. It would be helpful to quantify these phenotypes at the single cell level.*

We agree that the S6 Figure did not provide sufficient information on cell morphology. We now provided additional panels (see Fig R1-2 of this rebuttal letter and the new S6 Figure of the manuscript) for the distinct single cell morphologies of 1°NC and 2°NC cells.

Fig R1: Cell morphology of 1°NC and 2°NC cells. (E-F) A 18-hpf embryo from *Tg(sox10:mem-tdEosFP),* highlighting NC cells in green. (E) 3D-overview of the optic cup region (stippled circle). (F) Projected focal planes for the proximal side of the eye (stippled region). Note the cuboidal or hexagonal cell shape of 1°NC cells retaining large areas of cell-cell contact with neighbouring cells. (G-K) Overview of a 26-hpf *Tg(sox10:mem-tdEosFP)* embryo (G, maximum projection) and magnified single cell morphology of NC cells in a single focal plane for proximal (H-I) and distal (J-K) ocular NC cells. Squares in G show individual locations of magnified cells in H-K. 1°NC cells appear flat and rectangular (arrows), while 2°NC cells show mesenchymal morphology with filopodia-like cellular protrusions (arrowheads). Orientation: dorsal (D), ventral (V), rostral (R) and caudal (C); Ln: lens; Scale bars: 20 µm

We would like to point out S1 Figure D-F that shows the morphology of 1°NC cells retaining large cell-cell contact:

Fig R2: (D-F) Ultrastructure of 1°NC cells (magenta). (D) 1°NC cells are located on the proximal side of the optic cup (blue; transverse view at 18 hpf). The regions indicated by squares are shown in E and F. Black honey comb pattern is from the grid. (E) 1°NC cells form a mono-layered sheet. (F) A 1°NC cell (coloured in magenta) show a large cell-cell contact plane with neighbouring 1°NC cells. OSE: optic surface epithelium; Br: brain

In our current manuscript, the term “collective cell behaviour” appears only once in line 472 of the discussion:

We suspect that this reviewer dislikes this term and indeed we have no functional evidence on a collective phenomenon in the 1^o^NC cells. Since we discuss this solely on the basis of the distinct morphology of the migrating cells, we removed “collective”. We hope this satisfies this reviewer. *• p. 9, line 217-218, “Corneal endothelial cells proliferate until they occupy the entire inner surface of the cornea.” Did the authors show that proliferation is the driving mechanism?*

We do not know whether proliferation is the underlying mechanism. To be accurate, we replaced this sentence with: “The number of corneal endothelial cells increased and they eventually occupied the entire inner surface of the cornea.” *Reviewer #2: The authors have addressed all of my concern. Notably they are to be applauded for their extremely thorough rebuttal letter and many additional experiments included. Only one textual issue was apparent. In the morpholine experiments (S14), did they use published MO sequences. Perhaps this could be clarified in methods or in results section.*

Thank you for pointing this omission out. Morpholino experiments for revision were based on published sequences. We clarified this in the Methods section by adding references.

In addition, we would like to ask for your permission to add two missing information in the Methods: 1) line 640: an official file number for the animal experiment and 2) line 645: a reference article (Aleström et al. 2019)[reference 66] for zebrafish housing and husbandry.

**References**

Aleström, Peter, Livia D’Angelo, Paul J. Midtlyng, Daniel F. Schorderet, Stefan Schulte-Merker, Frederic Sohm, and Susan Warner. 2019. “Zebrafish: Housing and Husbandry Recommendations.” *Laboratory Animals*.

Reneker, Lixing W., Amy Bloch, Leike Xie, Paul A. Overbeek, and John D. Ash. 2010. “Induction of Corneal Myofibroblasts by Lens-Derived Transforming Growth Factor Β1 (TGFβ1): A Transgenic Mouse Model.” *Brain Research Bulletin*.
